# *“Pandemic Public Health Paradox”*: Time Series Analysis of the 2009/10 Influenza A / H_1_N_1_ Epidemiology, Media Attention, Risk Perception and Public Reactions in 5 European Countries

**DOI:** 10.1371/journal.pone.0151258

**Published:** 2016-03-16

**Authors:** Ralf Reintjes, Enny Das, Celine Klemm, Jan Hendrik Richardus, Verena Keßler, Amena Ahmad

**Affiliations:** 1 Hamburg University of Applied Sciences, Hamburg, Germany; 2 Radboud University Nijmegen, Nijmegen, the Netherlands; 3 VU University Amsterdam, Amsterdam, the Netherlands; 4 Erasmus MC, University Medical Center Rotterdam, Rotterdam, the Netherlands; 5 University of Tampere, Tampere, Finland; National Institute for Public Health and the Environment, NETHERLANDS

## Abstract

In 2009, influenza A H_1_N_1_ caused the first pandemic of the 21^st^ century. Although a vaccine against this influenza subtype was offered before or at the onset of the second epidemic wave that caused most of the fatal cases in Europe, vaccination rates for that season were lower than expected. We propose that the contradiction between high risk of infection and low use of available prevention measures represents a pandemic public health paradox. This research aims for a better understanding of this paradox by exploring the time-dependent interplay among changing influenza epidemiology, media attention, pandemic control measures, risk perception and public health behavior among five European countries (Czech Republic, Denmark, Germany, Spain and the UK). Findings suggest that asynchronicity between media curves and epidemiological curves may potentially explain the pandemic public health paradox; media attention for influenza A H_1_N_1_ in Europe declined long before the epidemic reached its peak, and public risk perceptions and behaviors may have followed media logic, rather than epidemiological logic.

## Introduction

In 2009, a new influenza A/H_1_N_1_ virus emerged causing the first pandemic of the 21^st^ century. The pandemic virus was first reported from Mexico on 4 April 2009, and spread rapidly across the globe [1]. Already on 27 April, the first laboratory confirmed cases were reported in Europe. A spring wave of transmission eventually followed by an autumn wave affected all EU countries [2–3]. Although a vaccine against this influenza subtype was offered in most of the European countries before, or at the onset of, the second epidemic wave that caused most of the fatal cases in Europe, vaccination rates were lower than expected, as a large proportion of citizens did not follow vaccination recommendations [4]. In some European countries vaccination rates against pandemic influenza were even lower than in previous years against seasonal influenza, even though the risk of infection in 2009 and 2010 was higher than during a normal influenza season. We propose that the contradiction between higher risk of infection and lower use of available prevention measures such as vaccination during the influenza A H_1_N_1_ pandemic represents a pandemic public health paradox.

The present research approaches the influenza A H_1_N_1_ pandemic from an interdisciplinary perspective in order to shed more light on this pandemic public health paradox. Building on theory and findings from epidemiology, communication science, and risk perception, this study examines the time-dependent interplay of the number of influenza A H_1_N_1_ cases, influenza A H_1_N_1_ casualties, media reports, pandemic control measures, risk perception and public health behavior across five European countries: Czech Republic, Denmark, Germany, Spain and the UK. Gaining convergent evidence across countries with different public health systems, media systems, and cultures is important to increase our understanding of generalizable patterns in epidemiology, media responses, and public responses, and the ways in which these patterns may interact.

Previous research has shown that influenza rates sometimes converge with (social) media patterns, but often times do not [5–7]. This lack of convergence may be explained by the fact that media logic does not equate epidemiological logic; for example, the first casualty in an epidemic has higher news value than later casualties, and influenza cases occurring close by have higher news value than cases occurring in a faraway country. News media can shape public perceptions in different ways, through (a) the sheer number of news reported in a specific time frame, (b) the content of media messages, and (c) the tone of coverage [8]. With regard to the content of media coverage of influenza A H_1_N_1_, evidence from existing media analyses indicates that information on the severity of and vulnerability to the influenza A H_1_N_1_ virus was the predominant theme in news reporting, followed by information of preventive measures (response efficacy) [9–13]. Tone of coverage was only examined by few studies. Most studies found little indication for alarmist, or dramatic reporting. For example, Duncan (2009) reported that 70% of articles published in European media during the first pandemic week were factual [14]. Hilton and Hunt (2011) likewise reported that the majority (83%) of articles over the further course of the pandemic had a factual, or neutral, tone [12]. In contrast, Vasterman and Ruigrok (2013) report that 74% of messages in The Netherlands contained alarming frames [15].

In the present research, we extend previous studies by zooming in on the amount of media coverage across different European countries during the entire influenza A H_1_N_1_ time span. Examining sheer media attention is important, as it determines not only what the public is pre-occupied with (agenda-setting function of media), it can also be a contributing factor to the social amplification–or attenuation—of risk [16–20], affecting risk perceptions and public behaviors. To date fairly little empirical evidence exists on this topic, except for the Netherlands [15], the UK [12], and the first week after the outbreak [14]. A review on worldwide news coverage of the influenza A H_1_N_1_ pandemic demonstrated that while media attention was large, it did not parallel epidemiological developments but rather was triggered mostly by key real-world events [8]. Findings across media analyses indicate that the volume of news coverage of the influenza A H_1_N_1_ pandemic was by far the highest at the very start of the pandemic but rapidly waned thereafter [11–15, 21], at trend that was similarly was found for online media [7, 22]. Other news peaks coincided with the official pandemic declaration on 11 June 2009 and a first wave of influenza A H_1_N_1_ cases, the start of a mass vaccination program, and an autumn wave of infections around October 2009 [11–12, 15, 22–23].

Because media attention may be related to public perceptions and behaviors, more so than epidemiological numbers [5], asynchronous media and epidemiological patterns may be one potential explanation for paradoxical public responses during epidemic outbreaks. The aim of the present research is to investigate explanations for the influenza A H1N1 case across different European countries. While previous research has examined, with mixed results, the relationship between actual influenza rates and (social) media attention or Google searches, systematic and detailed cross-national comparisons of epidemiological, media and behavioral data are still lacking.

## Methods

In order to analyse the time-dependent interplay between the different pandemic progress and control elements in five European countries—the Czech Republic (CZ), Denmark (DK), Germany (DE), Spain (ES) and the United Kingdom (UK)—different data sources were used.

### Epidemiology and control measures

A literature search was conducted using various combinations of the search terms “H_1_N_1_”, “epidemiology”, “population surveillance”, “prevalence“, “incidence”and “Europe” in Medline and Google Scholar. All articles about the influenza A H_1_N_1_ pandemic in the five selected countries published in English or German between 2009 and 2013 were included. In addition, reference lists of the retrieved articles, grey literature and websites of national health authorities (Robert Koch-Institute, Health Protection Agency, Spanish Ministry of Health and Social Policy, Statens Serum Institute and the Czech Ministry of Health), and international health agencies (European Centre for Disease Prevention and Control (ECDC), World Health Organization (WHO)) were searched. Data regarding the influenza A H_1_N_1_ epidemiology and surveillance, national public health measures taken, and the official health behavior recommendations given, were all retrieved for the time-period April 2009 to March 2010. For information on the reported number of influenza A H_1_N_1_ related deaths respective national health authorities were contacted if the information could not be found in published literature.

### Media attention

To investigate media attention between 1^st^ April 2009 and 31^rd^ March 2010 (1^st^ March 2009 to 28^th^ February 2010 for the UK), the volume of news reporting in selected print-media and TV newscasts was measured in the five countries. For DE, ES and CZ, two opinion-leading newspapers, a national evening newscast, and a weekly news magazine were selected (see Table 1 for the full media set). Media attention data for these three countries were obtained from the Swiss-based media research institute Media Tenor. For DK newspapers were retrieved from the LexisNexis database for one leading daily and one weekly newspaper. Data on the number of news articles in UK were obtained from Hilton and Hunt who published an analysis of UK news coverage during the influenza A H_1_N_1_ pandemic using eight newspapers [24]. Four opinion-leading newspapers based on category, political orientation and readership were selected (two daily fact led newspapers, one middle-market tabloid and one daily tabloid) (see Table 1).

**Table 1:** Media set used to determine media attention in the five studied countries.

	TV–Evening Newscast	Daily serious newspaper	Daily middle-market tabloid newspaper	Daily tabloid newspaper	Weekly newspaper
**Germany**	ARD Tagesschau	Frankfurter Allgemeine Zeitung		Bild	Spiegel
**Spain**	TVE Telediario	El Pais		20 minutos	Tiempo (online)
**Czech Republic**	CTV Udalosti	Lidove Noviny		Blesk	Respekt
**United Kingdom** *	-	(i) The Daily Telegraph*(ii) The Guardian*	The Daily Mail & Mail on Sunday*	The Su^n^*	-
**Denmark**	-	Politiken	-	-	Politiken Weekly

*Data on the number of news articles were obtained from Hilton and Hunt; 2011 [24]

The news items for DE, ES and CZ were identified using the search terms “H_1_N_1_ and/or Swine flu and/or new virus” in the respective languages in newspaper archives. All news items were retrieved, scanned for relevance and included if they referred primarily to influenza A H_1_N_1_, swine flu or swine flu vaccination. News stories were defined as primarily referring to influenza A H_1_N_1_, if the topic took up either (a) the greatest part, or (b) at least half of the news item (more than any other issue), or (c) was mentioned in the headline or (d) depicted in an illustration. Similarly, articles in the UK and the DK newspapers were retrieved and sorted based on the described inclusion criteria. Published letters to the editor were excluded.

### Risk perception and the public’s response

To assess public risk perception and vaccine uptake as a proxy for public reaction to the pandemic, national surveys conducted in DE and UK were included. Further information was obtained from the *Flash Eurobarometer–Influenza A H*_*1*_*N*_*1*_ *analytical report* which included 27 EU member states [25]. The national health authorities in DE, DK, and UK were contacted for additional information on vaccine uptake.

Time series analyses were undertaken based on a series of activities: the number of confirmed influenza A H_1_N_1_ cases and deaths; information on key events and pandemic control measures; vaccine uptake among the population over time, where available; and volume of media attention during the pandemic. The dynamics and interactions of these elements were analyzed and discussed in the context of current research.

## Results

**Fig 1:**
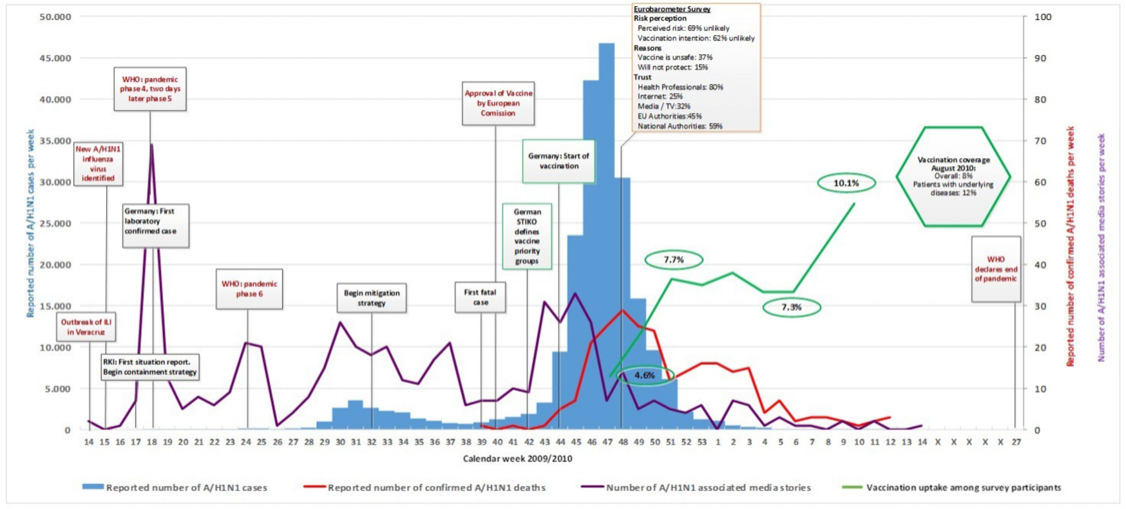
Epidemiology, key events and media attention during the A/H1N1 pandemic in Germany. Detailed data on the (i) number of new A/H1N1 cases per week, (ii) reported number of confirmed A/H1N1 deaths, (iii) media attention and (iv) vaccination uptake are provided in ‘S1 Data’. Data on vaccination coverage was taken from Mereckiene et al. [4] and data on risk perception from the Flash Eurobarometer survey [25]. Data on key events are provided in ‘S1 File’.

**Fig 2:**
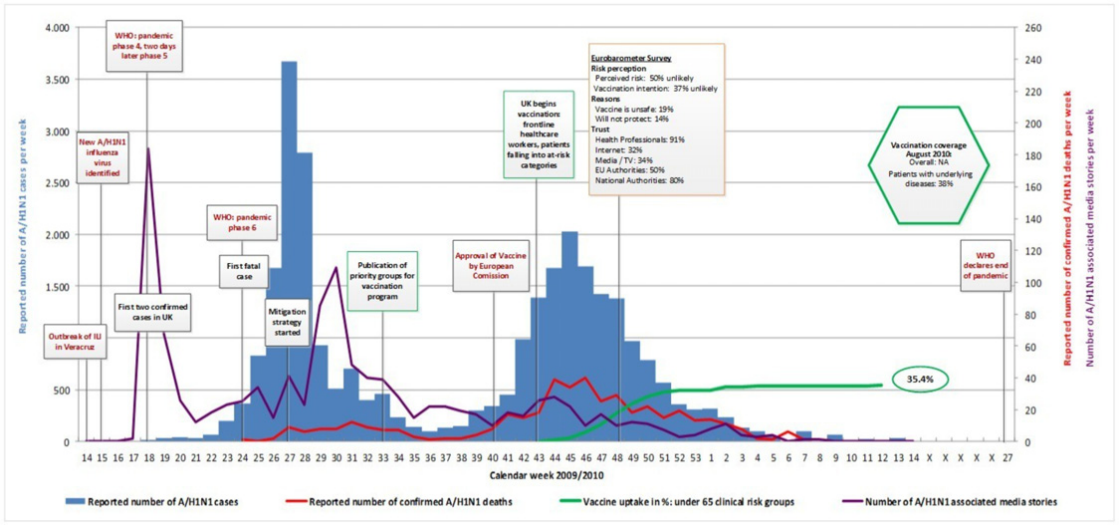
Epidemiology, key events and media attention during the A/H1N1 pandemic in UK. Detailed data on the (i) number of new A/H1N1 cases per week, (ii) reported number of confirmed A/H1N1 deaths, (iii) media attention and (iv) vaccination uptake are provided in ‘S1 Data’. Data on vaccination coverage was taken from Mereckiene et al. [4] and data on risk perception from the Flash Eurobarometer survey [25]. Data on key events are provided in ‘S1 File’.

**Fig 3:**
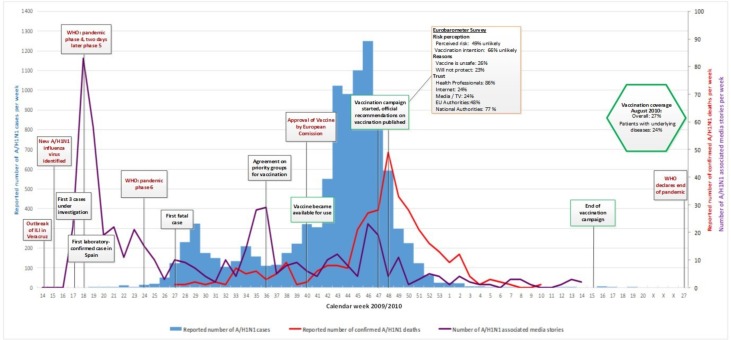
Epidemiology, key events and media attention during the A/H1N1 pandemic in Spain. Detailed data on the (i) number of new A/H1N1 cases per week, (ii) reported number of confirmed A/H1N1 deaths and (iii) media attention are provided in ‘S1 Data’. Data on vaccination coverage was taken from Mereckiene et al. [4] and data on risk perception from the Flash Eurobarometer survey [25]. Data on key events are provided in ‘S1 File’.

**Fig 4:**
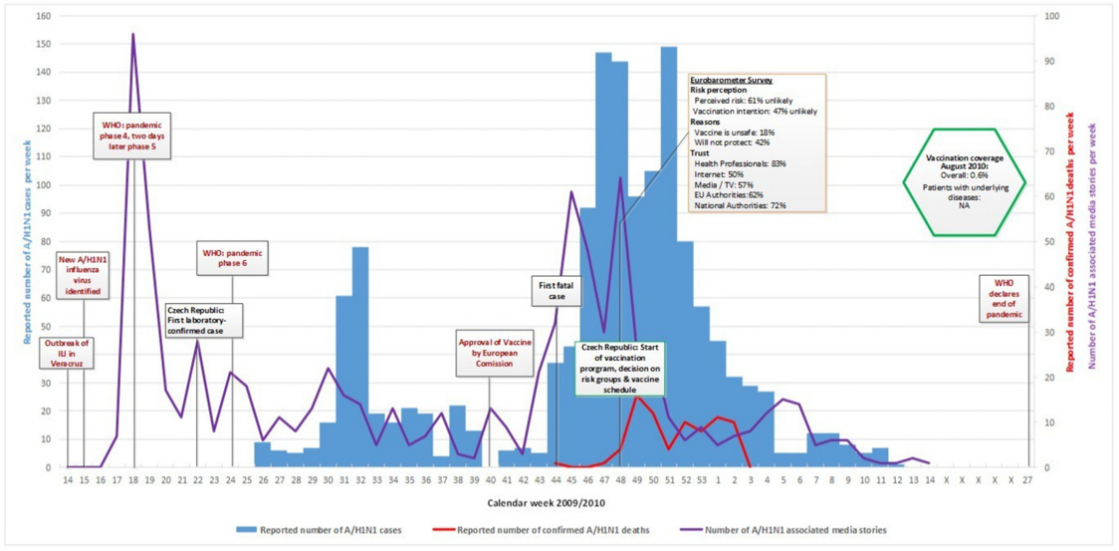
Epidemiology, key events and media attention during the A/H1N1 pandemic in Czech Republic. Detailed data on the (i) number of new A/H1N1 cases per week, (ii) reported number of confirmed A/H1N1 deaths and (iii) media attention are provided in the supplementary file ‘S1 Data’. Data on vaccination coverage was taken from Mereckiene et al. [4] and data on risk perception from the Flash Eurobarometer survey [25]. Data on key events are provided in ‘S1 File’.

**Fig 5:**
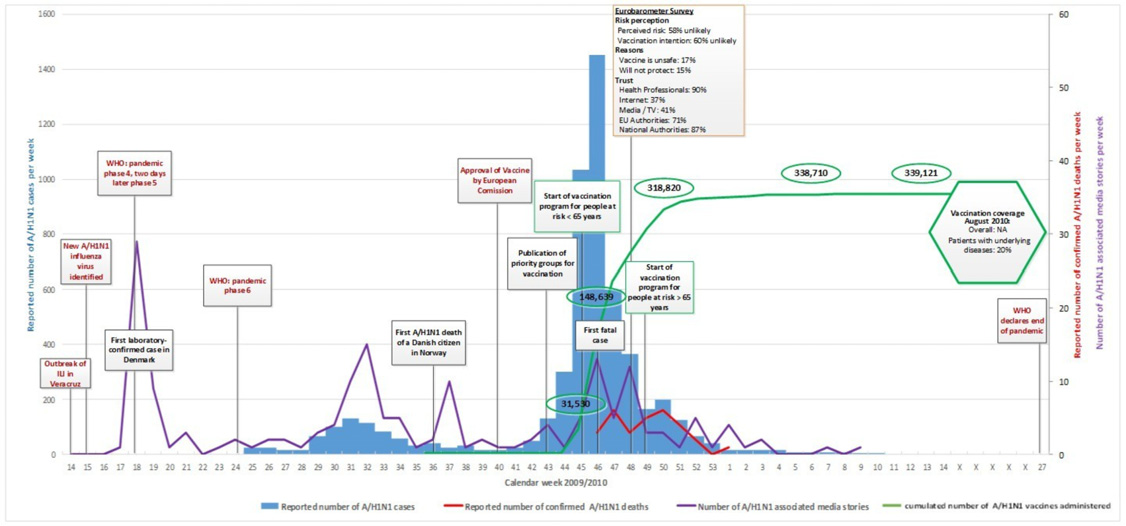
Epidemiology, key events and media attention during the A/H1N1 pandemic in Denmark. Detailed data on the (i) number of new A/H1N1 cases per week, (ii) reported number of confirmed A/H1N1 deaths, (iii) media attention and (iv) vaccination uptake are provided in the supplementary file ‘S1 Data’. Data on vaccination coverage was taken from Mereckiene et al. [4] and data on risk perception from the Flash Eurobarometer survey [25]. Data on key events are provided in‘S1 File’.

Figs 1–5 illustrate the results of the time series analysis in terms of the reported number of cases (blue bars, scales to left Y-axis), the reported number of confirmed influenza A H_1_N_1_ deaths (red curve, scales to right Y-axis) and the number of news reports / news items published (purple curve, scales to right Y-axis) for each country. The overall and risk-group specific vaccination coverage in August 2010 is shown in the green hexagon. The text in the rectangular boxes describes important milestones and key events which occurred both internationally (red text) or in the respective country (black text) during the course of the pandemic.

### Epidemiology

All five countries were significantly affected by two influenza A H_1_N_1_ waves–the first in spring followed by the second, larger wave in autumn/winter 2009 [Table 2]. However, during the course of the pandemic, some of the reporting requirements were changed. Thus, the actual number is likely to have been higher than reflected in Figs 1–5. Nonetheless, the epidemic curves still reflect the trends of influenza A H_1_N_1_ spread in the countries.

**Table 2:** Timing (calendar week in 2009) of key events related to the A/H1N1 pandemic (between April 2009 to March 2010), in the Czech Republic, Denmark, Germany, Spain and the UK (England); pertaining to the epidemiology (cases and deaths), media attention, vaccination and risk perception.

Country	A/H1N1 cases (calendar week in 2009)			A/H1N1 associated deaths (calendar week in 2009)		Media attention(calendar week in 2009)			A/H1N1 vaccination(calendar week in 2009)		A/H1N1 –Eurobarometer–Risk perception survey (conducted b/w 26^th^– 30^th^ Nov 2009
	1st A/H1N1 cases	1st peak	2nd peak	1st A/H1N1 deaths	Peak A/H1N1 deaths	1st peak	2nd peak	3rd peak	Start of vaccination	General Population coverage %	Perceived risk to get infected %Intention to get vaccinated %
**Czech Republic**	26*25*.*05*.*2009*	32	47–51	44	49	18	45	48	40	0.6%	Perceived risk: 61% unlikely Vaccination intention:47% unlikely
**Denmark**	18*01*.*05*.*2009*	31	45–46	46	47 & 50	18	31–32	46	45	N.A.	Perceived risk: 58% unlikely Vaccination intention: 60% unlikely
**Germany**	18*29*.*04*.*2009*	30–34	46–48	39	46–50	18	30	44–46	44	8%	Perceived risk: 69% unlikely Vaccination intention: 62% unlikely
**Spain**	19*27*.*04*.*2009*	29	43–46	27	48	18	35–36	46	40	27%	Perceived risk: 49% unlikely Vaccination intention: 66% unlikely
**United Kingdom / England**	18*27*.*04*.*2009*	26–28	43–48	24	44–46	18	30	43–44	43	N.A.	Perceived risk: 50% unlikely Vaccination intention: 37% unlikely

### Surveillance

The surveillance strategies of the observed countries were changed and adapted according to the varying epidemiology of the virus throughout the pandemic. On 16^th^ July, the WHO announced that countries with community-wide transmission are no longer required to forward regular reports of individual confirmed cases to the WHO. This was changed as the detection, laboratory-confirmation and investigation of all cases were extremely resource-intensive and not sustainable [26]. Therefore, the data quality and completeness of the reported influenza cases vary between countries and over time.

### Pandemic control measures

Initially DE, DK, ES and UK applied a containment strategy. Measures were focused on limiting transmission of the virus or delaying the spread in order to gain time to apply effective response measures. This strategy included laboratory confirmation of suspected cases, contact tracing and isolation of cases [27–30]. Further, all included countries recommended antiviral therapy within 48 hours after onset of symptoms [27–31] and requested persons with symptoms to be isolated at home or in hospital (depending on their clinical condition) for at least seven days [27–30, 32].

Widespread viral activity within the country, led UK to move from containment to treatment phase on 2^nd^ July 2009. Laboratory testing was no longer required for all cases and case-tracing was stopped. Further, antiviral treatment was only offered to clinical cases [33]. To relieve some of the pressures on the health system, the National Pandemic Flu Service was launched in England on 23^rd^ July. This was an online and telephone self-care service that allowed people outside the “at-risk” groups to be assessed for pandemic flu, and if required, to get access to antiviral treatment without the need to consult a physician [33].

Denmark moved to a mitigation strategy on 7^th^ July. The focus was on preventive treatment of persons at risk. Only risk group patients or persons with close contact to a risk group patient needed to be swabbed. Further, antiviral treatment was administered to risk group persons only, and prophylactic antiviral treatment was given to contacts of laboratory-confirmed cases only if the contact belonged to a risk group. This included persons with chronic pulmonary conditions, cardiovascular diseases, diabetes, immunodeficiency, HIV-Infection and pregnant women (2^nd^ and 3^rd^ Trimester). Furthermore, it was recommended that pregnant women in their 1^st^ trimester, children < 5 years and severely obese patients should be closely monitored [34, 35].

On 27^th^ July, Spain officially moved from containment to mitigation, although response measures had already changed towards mitigation in late June, i.e. contact tracing was ceased. Case-based reporting in the community was stopped, and antivirals were only given to cases requiring hospitalization and to those at risk of complications [36, 37].

From early August 2009, Germany applied a mitigation strategy, which predominantly focused on risk groups. In this strategy, contact-tracing was stopped. Isolation was recommended for cases with contact to vulnerable persons only. Antivirals were only given to cases in at-risk groups with signs of developing severe illness and case-based reporting requirements were relaxed [27].

Czech Republic started with a mitigation strategy on 9^th^ July [38]. Further details of the strategy could not be retrieved.

### Vaccination

In late September 2009 (week 40), the European Commission granted approval for two influenza A H_1_N_1_ vaccines, Focetria® (Novartis) and Pandemrix® (GlaxoSmithKline), in all EU Member States as well as Iceland, Liechtenstein and Norway [39]. The third vaccine for influenza A H_1_N_1_, Celvapan® (Baxter), was approved in early October 2009 [40]. All five study countries implemented a vaccination program around the time of the second wave (starting between week 40 to 44; week 48 in CZ) [Table 2] [4, 41–45] initially focusing on priority groups which in many cases was extended to the general public.

### Media attention

In all researched countries media attention, defined as the number of published news reports on influenza A H_1_N_1_, was highest in week 18 [Table 2], when the WHO declared pandemic phase 4 and shortly thereafter, pandemic phase 5. Media attention rapidly waned in all countries and was followed only by smaller peaks in news coverage over the remaining course of the pandemic Figs 1–5. Media attention curves differed among countries.

Germany: News reporting showed a small surge in media attention in week 24 coinciding with the WHO’s official pandemic declaration, and another peak in week 30 contemporaneous with the first wave of influenza A H_1_N_1_ transmission. The start of a third surge in attention corresponded with the official German definition of vaccine priority groups; its peak in week 43–45 paralleled with the start of the mass vaccination program.

United Kingdom: In week 28, after the first and largest wave of transmission, a second surge in media attention began peaking in week 30. Other smaller peaks coincide with the introduction of pandemic control measures (mitigation strategy; introduction of vaccination).

Denmark: After the first large peak in media attention, three smaller peaks could be observed. The first occurred in week 31–32 concurrent with the first wave of transmission, the second in week 37 following the first fatality abroad from Danish origin, and the third coinciding with the first national fatal case and the start of the mass vaccination program.

Spain: Following the initial peak, media attention was substantially lower over the remaining pandemic course than in the other countries. A smaller surge in media attention, coinciding with the agreement of priority groups for vaccination, began in week 33, peaking during weeks 35 to 36. It is notable that the peak began the week before the agreement, and ceased at approximately the time of agreement. In week 45 another peak emerged, which corresponded with a second wave in influenza A H_1_N_1_ transmission and the start of the national vaccination campaign.

Czech Republic: Media attention remained low until week 42, when two contiguous media attention peaks emerged. The first one, peaking in week 45, coincided with the first fatal case although not entirely triggered by it. The second one, peaking in week 48, corresponded with the start of the national mass vaccination program.

During the second wave of the epidemic, when most influenza-related deaths occurred, relatively little media attention was seen in all five study countries.

### Risk perception and vaccination uptake

In late November 2009, the Gallup Organization conducted a survey named Flash Eurobarometer in 30 European countries to assess public opinion about influenza and pandemic influenza A H_1_N_1_. In this survey, 69% of the German (N = 1001), 61% of the Czech (N = 1002), 58% of the Danish (N = 1008), and 49% of the UK participants (N = 1000) believed it was not at all likely or rather unlikely that they would personally catch the A/H_1_N_1_ influenza. The majority of the participants from ES (66%), DE (62%) and DK (60%) also stated that is was not likely or not at all likely that they would get vaccinated against the pandemic A/H_1_N_1_ virus. This proportion was considerably lower in the UK (37% of the participants) and the CZ (47% of the Czech interviewees) [Table 2] [25].

For the included countries, the vaccination coverage of persons with underlying diseases as well as the overall uptake, if available [4], is shown in the green hexagon in Figs 1–5.

In the UK, the vaccine uptake in clinical risk groups was assessed using data collected from a sentinel group of GP practices in England. The vaccination uptake among the “under 65” clinical risk groups is reflected in the green curve in Fig 2. In this group as well as in the over 65 years age group (curve not shown), the vaccine uptake increased steadily until week 4/2010. Overall, the national vaccine uptake in patients in clinical risk groups aged under 65 years was 35.4%, this included pregnant women. It was 40.4% in those aged 65 years and over. Another survey assessed the vaccine uptake among healthcare workers in all 389 NHS Trusts in England on a weekly basis from 8^th^ November 2009 to 4^th^ April 2010. The vaccine uptake among healthcare workers increased sharply in the first weeks after the vaccine was available and leveled out at approximately 40% from week 4 of 2010 [46].

For DK, the green curve in Fig 5 shows the number of vaccines administered per week during the pandemic. In week 45, when the vaccination program for people at risk started, 31,530 doses were administered. For people at risk > 65 years the vaccination began in week 49 and 50, the vaccine doses administered increased sharply to 318,820 doses before leveling out thereafter.

The pandemic influenza A H_1_N_1_ vaccine uptake during the vaccination campaign in DE was monitored using 13 bi-weekly telephone-surveys between November 2009 and April 2010. Among the survey participants (approximately 1,000 households per survey), the vaccination coverage in persons ≥ 14 years of age was 4.6% in week 47, 6% in week 49, 7.3% to 7.9% until week 6 and reached 10.1% in week 10 in 2010 [47].

The overall and group-specific vaccination coverage cannot be compared between the countries due to methodological differences in its assessment. Nonetheless, the trends in vaccine uptake over time indicate similarities in UK, DK and DE.

The vaccine uptake throughout the pandemic could not be illustrated for CZ and ES since data could not be obtained for these two countries.

## Discussion

The combined analysis of the elements described above, confirms that media logic does not equate to epidemiological logic, which adds to prior—yet mixed—findings that influenza rates oftentimes diverge from (social) media patterns (e.g., Mollema et al., 2015; Fenichel et al., 2013; Signorini et al. 2011) [5–7]. In the case of the 2009 influenza A H_1_N_1_, media attention did not increase with increased number of infections or casualties but rather spotlighted certain key events based on their news value. This pattern was consistently found across all five European countries, indicating its generality beyond national media systems and cultures. Media attention surged with the WHO declarations of a pandemic (in week 18), before the pandemic started to spread across European countries, and dropped to lower levels after this first peak. Later on, the media did respond to news worthy real world events such as the introduction of the vaccine and the first nationally reported death (in most but not all countries), however media patterns show little concurrence with the number of cases and the number of confirmed deaths across countries [Table 2].

Google Flu Trends analysis of influenza-related web search queries in Germany and Spain showed that, in comparison, search activity for influenza-like disease increased significantly later in the course of the pandemic and reflected the actual epidemic curve peaks in Germany and Spain fairly well. This indicates that public interest in influenza related information increased with rising influenza cases in the own country [48]. Since there is a reporting lag of only one day and the search queries can be analyzed quickly, this may be a valuable information source for an up-to-date disease activity trend [49].

Although we cannot draw definitive conclusions regarding causal patterns, the finding that the media curves declined long before the epidemiological curves, paired with the established knowledge that the sheer amount of news reports can contribute to the social amplification or attenuation of risk [16–20], could be one of the potential reasons for the rather low risk perception of citizens with respect to influenza A H_1_N_1_ and vaccination uptake across countries. Apart from sheer media attention, media content may also play a role in explaining the pandemic public health paradox [50]. For example in Germany there was a period of media discourses questioning the safety of the vaccine and the availability of a different vaccine for the military and government officials in contrast to the general population was high on the media agenda. Media attention to barriers for vaccination, such as vaccine safety, may have had negative impact on public perception of the vaccine and vaccination rates.

Apart from the content, the magnitude of attention, an issue arouses in influenced by the frequency with which this issue is communicated and repeated in different media sources. A time-series analysis of 36 telephone surveys in the UK revealed that the level of worry of contracting swine-flu (which was quite low (9.6% to 32.9%)) was associated with the volume of media messages during the first summer wave of the epidemic in the UK but not prior to community transmission in the UK or during the autumn/winter wave. The study also showed that a level of higher worry of oneself of one’s child getting infected was associated with a higher vaccine acceptance. The authors conclude that when levels of worry are generally low, a rise in the frequency of media messages could increase the public’s perception in the effectiveness and therefore the uptake of protective behavior [51]. In our study, the media attention was lower in all five countries when sustained in-country transmission was going on compared to the early phase. This paired with lower levels of worry is likely to reduce vaccine uptake.’

Utmost care was taken to use comparable data sources for the five study countries and to transparently describe where the data was retrieved from and which data was not available. We feel that even if the choice of data sources may have some effect on the comparability in terms of absolute numbers, the trend captured is comparable.

The observed divergence of media attention and disease epidemiology poses a challenge for countries in such an epidemic situation. Firstly, it is very unlikely, that sufficient information about a new virus or an unusual strain of a virus, its potential spread and the disease’s severity is known early in a pandemic. At this stage generally only likely projections can be made about the course of the pandemic and containment measures initiated, but this is the period when the media’s spotlight is on and the issue is highest on the public agenda [19].

Using the initial media attention peak in week 18 of 2009 to inform the population about the virus, the potential spread in the country, as well as recommended preventive and curative measures was rather difficult with missing facts. Media spotlights, even if preceding events in the own country, offer windows of opportunity to inform the public about resources where they can seek reliable information when it becomes available and when public interest rises. One can thus establish a channel for communication that remains even after the media spotlight has moved on to other issues. This is very important considering that media follows different logic, hence it can be expected that coverage of epidemiological facts with lower news values but high importance for public health will only be limited.

Our analysis further shows that the main vaccination uptake across countries occurred within a short period of around four weeks after the start of vaccination. This short time-span could likewise be regarded as a window of opportunity. Concerted efforts should be undertaken to avoid a “pandemic public health paradox” in future epidemic situations. These efforts should be made by state ministries, public health institutes and especially by health professionals, who are regarded as the most trusted source of information, according to the Eurobarometer survey [25]. Public health officials need to be aware of the different media logic and the short periods of media spotlights and use them appropriately in order to provide the right information to their audience at the right time.

## Supporting Information

S1 Data Influenza A-H1N1 combined dataset—CZ DE DK ES and UK—(used for the time-series analysis). (XLSX)

S1 File Chronology of Key Events during the A-H1N1 Pandemic in CZ DK DE ES and UK plus References. (PDF)

## References

[1] European Centre for Disease Prevention and Control. European 2009 Influenza Pandemic Timeline. 2010. Available: http://ecdc.europa.eu/en/healthtopics/H1N1/Documents/110810_2009_pandemic _European_Timeline.pdf.

[2] European Centre for Disease Prevention and Control. Situation Report—Infections of novel flu virus (A/H1N1). 2009. Available: http://ecdc.europa.eu/en/healthtopics/Documents/090428_InfluenzaAH1N1_Situation_Report_0800hrs.pdf.

[3] World Health Organization. Pandemic (H1N1) 2009—update 66. 2009. Available: http://www.who.int/csr/disease/swineflu/laboratory18_09_2009/en/.

[4] Mereckiene J, Cotter S, Weber JT, Nicoll A, D'Ancona F, Lopalco PL, et al Influenza A(H1N1)pdm09 vaccination policies and coverage in Europe. Eurosurveillance 2012; 17(4). 10.2807/ese.17.04.20064-en 22297139

[5] Mollema L., Harmsen I.A., Broekhuizen E., Clijnk R., De Melker H., Paulussen T. et.al Disease detection or public opinion reflection? Content analysis of tweets, other social media, and online newspapers during the measles outbreak in the Netherlands in 2013. Journal of Medical Internet Research 2015, 17(5), e128 10.2196/jmir.3863 26013683 PMC4468573

[6] Fenichel EP, Kuminoff NV, Chowell G. Skip the Trip: Air Travelers’ Behavioral Responses to Pandemic Influenza. Boni MF, ed. PLoS ONE. 2013;8(3):e58249 10.1371/journal.pone.0058249 23526970 PMC3604007

[7] Signorini A., Segre A. M., Polgreen P. M. The use of Twitter to track levels of disease activity and public concern in the U.S. during the influenza A H1N1 pandemic. PloS One 2011, 6(5), e19467 10.1371/journal.pone.0019467 21573238 PMC3087759

[8] Klemm C., Das E., Hartmann T. Swine Flu and Hype: A Systematic Review of Media Dramatization of the H1N1 Influenza Pandemic. Journal of Risk Research 2014.

[9] Chang C. News coverage of health-related issues and its impacts on perceptions: Taiwan as an example. Health Communication 2012, 27(2), 111–23. 10.1080/10410236.2011.569004 21843098

[10] Fogarty A. S., Holland K., Imison M., Blood R. W., Chapman S., Holding S. Communicating uncertainty-how Australian television reported H1N1 risk in 2009: a content analysis. BMC Public Health 2011, 11, 181 10.1186/1471-2458-11-181 21435263 PMC3079644

[11] Goodall C., Sabo J., Cline R., Egbert N. Threat, Efficacy, and Uncertainty in the First 5 Months of National Print and Electronic News Coverage of the H1N1 Virus. Journal of Health Communication 2012, 17(3), 338–55. 10.1080/10810730.2011.626499 22188164

[12] Hilton S., Hunt K. UK newspapers’ representations of the 2009–10 outbreak of swine flu: one health scare not over-hyped by the media? Journal of Epidemiology and Community Health 2011, 65(10), 941–946. 10.1136/jech.2010.119875 21131303 PMC3171979

[13] Yu N., Frohlich D. O., Fougner J., Ren L. Communicating a Health Epidemic: A Risk Assessment of the Swine Flu Coverage in US Newspapers. International Public Health Journal 2011, 3(1 (Special Issue on “Health Risk Communication”), 63–76

[14] Duncan B. How the media reported the first days of the pandemic (H1N1) 2009: results of EU-wide media analysis. EuroSurveillance 2009, 14(30), 1–3. Available: http://www.eurosurveillance.org/ViewArticle.aspx?ArticleId=19286. 10.2807/ese.14.30.19286-en 19643058

[15] Vasterman P. L., Ruigrok N. Pandemic alarm in the Dutch media: Media coverage of the 2009 influenza A (H1N1) pandemic and the role of the expert sources. European Journal of Communication 2013, 28(4), 436–453. 10.1177/0267323113486235

[16] Fischhoff B. Managing risk perceptions. Issues in Science and Technology 1985; 2: 83–96.

[17] Fischhoff B. Risk perception and communication unplugged; Twenty years of progress. Risk Analysis 1995; 15(2):137–145. 7597253 10.1111/j.1539-6924.1995.tb00308.x

[18] Kitzinger J, Reilly J. The rise and fall of risk reporting. Media coverage of human genetics research, ‘false memory syndrome’ and ‘Mad Cow Disease’. European Journal of Communication 1997; 12:319–50

[19] McCombs ME, Shaw DL. A progress report on agenda-setting research. Paper presented at the Association for Education in Journalism, San Diego, CA, USA 1974

[20] Kasperson RE, Renn O, Slovic P, Brown J, Emel R Goble JX et al The Social Amplification of Risk: A conceptual Framework. Risk Analysis 1988; 8(2):177–87

[21] Tausczik Y., Faasse K., Pennebaker J. W., Petrie K. J. Public anxiety and information seeking following the H1N1 outbreak: blogs, newspaper articles, and Wikipedia visits. Health Communication 2012, 27(2), 179 Pub 10.1080/10410236.2011.571759 21827326

[22] Chew C., Eysenbach G. Pandemics in the age of Twitter: content analysis of Tweets during the 2009 H1N1 outbreak. PloS One 2010, 5(11), e14118 10.1371/journal.pone.0014118 21124761 PMC2993925

[23] Rachul C.M., Ries N.M., Caulfield T. Canadian newspaper coverage of the A/H1N1 vaccine program. Canadian Journal of Public Health. Revue Canadienne de Santn Publique 2011, 102(3), 200(203. Available: http://journal.cpha.ca/index.php/cjph/article/view/2493 10.1007/BF03404896 PMC6973664 21714319

[24] Hilton S, Hunt K. UK newspapersp/cjph/article/view/2493le/view/2493" ournal of Public Health. Revue Canadienne de Santnalth Communication 1988; 8(2):177-874pean Journal of Communication 1997; 12:319

[25] The Gallup Organization. Flash Eurobarometer 287. Eurobarometer on Influenza H1N1. 2010. Available: http://ec.europa.eu/public_opinion/flash/fl_287_en.pdf.

[26] World Health Organization. Changes in reporting requirements for pandemic (H1N1) 2009 virus infection. 2009. Available: http://www.who.int/csr/disease/swineflu/notes/h1n1_surveillance_20090710/en.

[27] Robert Koch-Institut. Rückblick: Epidemiologie und Infektionsschutz im zeitlichen Verlauf der Influenzapandemie (H1N1) 2009. Epidemiologisches Bulletin 2010; (21):191–197.

[28] Health Protection Agency. Epidemiological report of pandemic (H1N1) 2009 in the UK. April 2009—May 2010. 2010. Available: http://www.hpa.org.uk/webc/HPAwebFile/HPAweb_C/1284475321350.

[29] Santa-Olalla Peralta P, Cortes García M, Martínez Sánchez EV, Nogareda Moreno F, Limia Sánchez A, Pachón del Amo I, et al Vigilancia individualizada de los casos iniciales de infección por gripe pandémica (H1N1) 2009 en España, abril-junio 2009. Revista Española de Salud Pública 2010; 84(5):529–46. 21203718 10.1590/s1135-57272010000500007

[30] Andersen PH. Influenza A/H1N1 of new subtype (swine influenza). EPI-News. National Surveillance of Communicable Diseases 2009 2013/03/10(18).

[31] Ministerstvo zdravotnictví ÈR. Léky Tamiflu–aktualizace údajù pro zdravotníky. 2009. Available: http://pandemie.mzcr.cz/Pages/285-tamiflu-aktualizace-udaju-pro-zdravotniky.html.

[32] Ministerstvo zdravotnictví ÈR. Pro cestovatele 21.5.09—Aktualizavané Doporuèení Ministerstva zdravotnictví pro cestovatele. 2009. Available: http://pandemie.mzcr.cz/Pages/104-21509-aktualizavane-doporuceni-ministerstva-zdravotnictvi-pro-cestovatele.html.

[33] Hine DD. The 2009 Influenza Pandemic. An independent review of the UK response to the 2009 influenza pandemic. 2010. Available: http://www.dhsspsni.gov.uk/the2009influenzapandemic_acc.pdf.

[34] Andersen PH. Influenza A (H1N1) clarification of new guidelines. EPI-News. National Surveillance of Communicable Diseases 2009 2013/03/10(27–29).

[35] National Board of Health. Ændret strategi for håndtering af influenza A (H1N1). 2009 06/07; 2013/03/05.

[36] Sierra Moros J, Vázquez Torres M, Santa-Olalla Peralta P, Limia Sánchez A, Crtes Garcia M, Pachón dA, I. Actividades de vigilancia epidemiológica durante la pandemia de Gripe (H1N1) 2009 en España. Reflexiones un año despuás. Revista Española de Salud Pública 2010; 84(5):463–79. 21203713 10.1590/s1135-57272010000500002

[37] Ministerio de Sanidad Politica Social e Igualdad. Análisis de la actuación en materia de vacunas y antivirales durante la pandemia de Gripe por virus A (H1N1)2009. 2010. Available: http://www.mspsi.gob.es/profesionales/saludPublica/gripeA/docs/informeSVAdic2010.pdf.

[38] European Centre for Disease Prevention and Control. Surveillance Report. Pandemic (H1N1) 2009 Weekly report: Individual case reports EU/EEA countries 31 July 2009. Available: http://ecdc.europa.eu/en/healthtopics/Documents/090731_Influenza_A%28H1N1%29_Analysis_of_individual_data_EU_EEA-EFTA.pdf.

[39] European Commission. Commission paves the way for vaccinations for influenza pandemic (H1N1) 2009. 2009. Available: http://europa.eu/rapid/press-release_IP-09-1384_en.htm?locale=en.

[40] European Commission. Midday Express. News from the Communication Directorate General`s midday briefing 2009. Available: http://europa.eu/rapid/press-release_MEX-09-1007_en.htm?locale=en.

[41] Department of Health. The H1N1 swine flu vaccination programme 2009–2010. 2009. Available: http://webarchive.nationalarchives.gov.uk/20130107105354/http://www.dh.gov.uk/prod_consum_dh/groups/dh_digitalassets/@dh/@en/documents/digitalasset/dh_107190.pdf.

[42] Bundesministerium für Gesundheit. Informationsangebot des Bundesgesundheitsministeriums zum Start der Impfungen gegen die Neue Grippe. 2009. Available: http://www.bmg.bund.de/fileadmin/redaktion/pdf_pressemeldungen/2009/091014-PM.pdf.

[43] Ministerio de Sanidad y Politica Social. Swine Flu Vaccination 2009. 2009. Available: http://www.informaciongripea.es/descargas/fichas/fase2/FICHA_VACUNACION_INGLES_baja.pdf.

[44] National Board of Health. Bestil tid til influenza A (H1N1) vaccination. 2009. Available: http://www.sst.dk/Nyhedscenter/Nyheder/2009/Vaccination_bestil_tid_65.aspx.

[45] O'Flanagan D, Cotter S, Mereckiene J. Pandemic A(H1N1) 2009 Influenza Vaccination Survey, Influenza season 2009/2010.VENICE II Consortium August 2010-April 2011. 2011. Available: http://venice.cineca.org/Final_Report_VENICE_Pandemic_Influenza_2009.pdf.

[46] Sethi, M., Pebody, R. Pandemic H1N1 (Swine) Influenza Vaccine Uptake amongst Patient Groups in Primary Care in England. 2010. Available: https://www.gov.uk/government/uploads/system/uploads/attachment_data/file/215977/dh_121014.pdf.

[47] Walter D, Böhmer MM, Heiden Ma, Reiter S, Krause G, Wichmann O. Monitoring pandemic influenza A(H1N1) vaccination coverage in Germany 2009/10—results from thirteen consecutive cross-sectional surveys. Vaccine 2011; 29(23):4008–12. 10.1016/j.vaccine.2011.03.069 21463683

[48] Google Flu Trends. Available: http://www.google.org/flutrends/intl/en_gb/.

[49] Ginsberg J, Mohebbi MH, Patel RS, Brammer L, Smolinski MS, Brilliant L. Detecting influenza epidemics using search engine query data. Nature 2008; 457(7232):1012–4. 10.1038/nature07634 19020500

[50] Klemm C, Das E, Hartmann T. (2014). Swine Flu and Hype: A Systematic Review of Media Dramatization of the H1N1 Pandemic. Paper presented at the ICA Conference, Seattle, USA.

[51] Rubin GJ, Potts HW, Michie S. The impact of communications about swine flu (influenza A H1N1v) on public responses to the outbreak: results from 36 national telephone surveys in the UK. Health Technol Assess. 2010 7;14(34):183–266. 10.3310/hta14340-03 20630124

