# Supplementary material for: “Pandemic Public Health Paradox”: Time Series Analysis of the 2009/10 Influenza A / H1N1 Epidemiology, Media Attention, Risk Perception and Public Reactions in 5 European Countries
Source: PLoS One. 2016 Mar 16;11(3):e0151258. doi: 10.1371/journal.pone.0151258 (PMC4794201; doi:10.1371/journal.pone.0151258)
Supplement: S1 File — (PDF) [file pone.0151258.s002.pdf]

Chronology of key events during the 2009 A/H1N1 pandemic in Czech Republic

Month	Day	Event
May	21	Travel recommendations and recommendations on protective measures (Ministerstvo zdravotnictví ČR, 2009a)
	25	First laboratory-confirmed case in Czech Republic (European Centre for Disease Prevention and Control, 2009q)
July	31	Information on Relenza for health professionals published (Ministerstvo zdravotnictví ČR, 2009b)
August	21	Purchase agreement between GSK and Czech Republic to buy 1 million doses of Pandemrix. First delivery can be expected in week 48/2009 (Ministerstvo zdravotnictví ČR, 2009c)
	27	Questions and answers based on ECDC material published (Ministerstvo zdravotnictví ČR, 2009d)
September 2009	16	Poster on preventive measures (Ministerstvo zdravotnictví ČR, 2009e)
	23	Information on antivirals for health professionals published (Ministerstvo zdravotnictví ČR, 2009f)
October 2009	26	First fatal case was reported in the Czech Republic (European Centre for Disease Prevention and Control, 2009af)
November 2009	16	Information material on vaccine and Tamiflu sent to GP (Ministerstvo zdravotnictví ČR, 2009g)
	20	Information on use and dosage of Tamiflu for hospitals published (Ministerstvo zdravotnictví ČR, 2009h)<br>Information on vaccine for vaccination centers published (Ministerstvo zdravotnictví ČR, 2009i)
	23	Czech Republic started vaccination program (O'Flanagan et al..,

		2011)
	25	Decision on risk groups and vaccine schedule published (Ministerstvo zdravotnictví ČR, 2009j)
	Week 48	Survey result: 61% (N=1002) believed it was not likely at all or rather unlikely that they would personally catch the A/H1N1influenza; 47% stated it was not likely at all or not likely that they would get vaccinated against pandemic A(H1N1).<br>83% (N=1002) perceived Health professionals to be the most trusted source of information (The Gallup Organization, 2010)
**December 2009**	4	Reported deaths in the Czech Republic: 22 (European Centre for Disease Prevention and Control, 2009ai)
	9	Questions and answers on vaccination based on ECDC material published (Ministerstvo zdravotnictví ČR, 2009k)
	11	Number of reported pandemic A/H1N1 deaths: 34 (European Centre for Disease Prevention and Control, 2009aj)
	28	Reported deaths in the Czech Republic: 48 (European Centre for Disease Prevention and Control, 2009al)
**January 2010**	15	Number of reported pandemic A/H1N1 deaths: 83 (European Centre for Disease Prevention and Control, 2010c)
**February 2010**	19	Number of reported pandemic A/H1N1 deaths: 96 (European Centre for Disease Prevention and Control, 2010d)
**March 2010**	19	Number of reported pandemic A/H1N1 deaths: 98 (European Centre for Disease Prevention and Control, 2010e)
**August 2010**	Aug	Vaccination coverage:<br>General population 0,6%<br>Healthcare workers 7%<br>Pregnant women 0% (Mereckiene et al.., 2012)

Chronology of key events during the 2009 A/H1N1 pandemic in Denmark

Month	Day	Event
**April 2009**	29	Advice against all unnecessary travel to Mexico (National Board of Health, 2009a)
		Department of Epidemiology published a guideline on the handling of suspected patients (Andersen, 2009a)<br>Containment strategy (National Board of Health, 2009h)
**May 2009**	1	First laboratory confirmed A/H1N1 case in Denmark (National Board of Health, 2009b)
		Information hotline was set up (National Board of Health, 2009e)
	18	Advice against travel to Mexico was lifted (National Board of Health, 2009f)
		Due to the novel influenza virus A(H1N1) outbreaks, the sentinel surveillance of influenza in Denmark was maintained beyond the normal influenza season (Andersen, 2009b)
**June 2009**	11	Total number of cases: 11, all of which have been relatively mild. Infection in Denmark is still limited to persons who have been abroad and in some cases their immediate contacts (National Board of Health, 2009g)
**July 2009**	6	Board of Health has decided to change its strategy for dealing with influenza A (H1N1) from 7 July 2009. Concentrating now on the treatment of those who are at risk and preventive treatment for people at risk (Andersen, 2009c; National Board of Health, 2009h)
	15	Notification regulation of suspected cases revised. The

		individual notification of suspected cases has been lifted and replaced by mandatory laboratory notification. The voluntary sentinel surveillance in primary health care which comprises submission of weekly reports and samples will be in place throughout the year (Andersen, 2009c)
**September 2009**	3	First death of a Danish citizen in Norway (National Board of Health, 2009i)
**October 2009**	1	Guidance for physicians and other health professionals on prevention measures and treatment of cases was published (National Board of Health, 2009k,2009j)
	21	Department of Epidemiology published questions and answers on pandemic vaccine (Pandemrix) (Andersen, 2009d)
	23	National Board of Health recommendations on priority groups for vaccination were published (National Board of Health, 2009m, 2009n)
**November 2009**	8	Denmark begins vaccination: people at risk who are under 65 years of age (National Board of Health, 2009l; O'Flanagan, Cotter, & Mereckiene, 2011)
	Week 46	Active reporting of influenza patients from all Intensive Care Units (ICUs) between week 46, 2009, and week 11, 2010 (Mølbak et al.., 2011).
	11	Surveillance strategy revised (Andersen, 2009e)
	Week 48	Survey result: 58% (N=1008) believed it was not likely at all or rather unlikely that they would personally catch the A/H1N1influenza; 60% stated it was not likely at all or not likely that they would get vaccinated against pandemic A(H1N1)

		90% (N=1008) perceived Health professionals to be the most trusted source of information (The Gallup Organization, 2010)
	30	Information leaflet and radio spots on influenza A/H1N1 in English and six widely used minority languages were published (Arabic, Urdu, Bosnian, Turkish, Somali, Persian) (National Board of Health, 2009c, 2009d, 2009o)
**December 2009**	Early Dec.	From the beginning of December vaccination of people at risk who are over 65 years old started (National Board of Health, 2009l)
	2	Board of Health adjusted vaccination recommendations. From now on, only one dose of vaccine for patients at risk, unless they have a weakened immune system (Andersen, 2009f; National Board of Health, 2009p)
		By the end of week 48, the SSI had distributed nearly 500,000 vaccine doses, primarily to cover risk group vaccination (Andersen, 2009f)
	9	Number of reported deaths in Denmark : 16, including three outside risk groups (National Board of Health, 2009q)
	18	A/H1N1 hotline was closed down (National Board of Health, 2009r)
**January 2010**	6	Total number of confirmed cases: nearly 5.000<br>Total number of deaths: 30<br>Hospitalizations: 1.000 (Andersen, 2010b)
**February 2010**	12	The government has decided to offer the excess influenza A (H1N1) vaccines for the citizens who have not already been offered the vaccine (National Board of Health, 2010)
**June 2010**	9	Total number of deaths: 32 - mainly among persons with

		underlying risk factors.<br>Total number of vaccinated persons: 339,515, including 286,568 with chronic illness, 5,780 pregnant women and 4,073 contacts to severely immunosuppressed patients. A total of 42,859 persons from the groups of healthcare professionals and key personnel were vaccinated (Andersen, 2010a)
**August 2010**	Aug	Vaccination coverage:<br>Chronic disease (<65) 20% (Mereckiene et al.., 2012)

ECOM

Chronology of key events during the 2009 A/H1N1 pandemic in Germany

Month	Day	Event
**March 2009**		RKI and BZgA: Information campaign “Wir gegen Viren” was developed and launched (Martin, 2010)
**April 2009**	4	Outbreak of influenza-like illness started in Veracruz, Mexico (European Centre for Disease Prevention and Control, 2010a)
	15	Novel Influenza A/H1N1 identified and isolated in USA (Centers for Disease Control and Prevention, 2009)
	24	WHO confirmed that the outbreak in Mexico was caused by a novel influenza virus (World Health Organization, 2009b)
		RKI: Teleconference with infectious disease experts of the 16 German states to assess the current situation and discuss relevant infection control measures (Robert Koch-Institute, 2010a)
	25	WHO declared the outbreak of influenza A/H1N1 in Mexico and the US as a Public Health Emergency of International Concern (PHEIC) under IHR (2005) (World Health Organization, 2009c)
		RKI: First situation report (Daily report, published until 4.12.2009) (Robert Koch-Institute, 2009b)
	26	RKI distributed information on surveillance and control to local health authorities. The strategy was to contain the spread of the virus (Robert Koch-Institute, 2010a)
	27	Local health authorities started infection control measures at airports and distributed information leaflets for travelers (Robert Koch-Institute, 2010a)
		RKI set up an information hotline for the general public (Robert

		Koch-Institute, 2010a)
		WHO declared influenza pandemic alert phase 4 (World Health Organization, 2009d)
		First laboratory confirmed case of A/H1N1 announced in Europe. One in Spain and two in the UK (European Centre for Disease Prevention and Control, 2009f)
	29	WHO declared influenza pandemic alert phase 5 (World Health Organization, 2009e)
		RKI reported first confirmed cases in Germany (Robert Koch-Institute, 2009h)
	30	The European Union agreed on a common case definition for the new pandemic virus (European Commission, 2009a)
**May 2009**	1	First case of secondary transmission in the UK and Germany (European Centre for Disease Prevention and Control, 2009i)
		Free information hotline for the general public set up by Ministry of Health (Robert Koch-Institute, 2010a)
	3	5 cases of in country transmissions in Germany, Spain and UK (European Centre for Disease Prevention and Control, 2009j)
		From week 18 onwards notification regulation for all suspected cases and deaths (Bundesministerium der Justiz, 2009)
**June 2009**	11	WHO raised the level of influenza pandemic alert from phase 5 to phase 6 (World Health Organization, 2009h)<br>WHO considered severity of pandemic to be moderate (World Health Organization, 2009i)
**July 2009**	13	Notification regulation of suspected cases revised. From week 29 onwards suspected cases did not have to be reported to state health authorities or RKI anymore; only to regional health

		authorities (Robert Koch-Institute, 2010a)
	15	Information campaign started (Die Beauftragte der Bundesregierung für Migration, Flüchtlinge und Integration, 2009)
**August 2009**	Early	Mitigation phase began (Robert Koch-Institute, 2010a)
	17	Statutory health insurances paid costs for laboratory confirmation only for cases with severe disease or cases at risk of developing severe disease (Gilsdorf & Poggensee, 2009)
	24	In week 35 infection control measures at airports were reduced (Robert Koch-Institute, 2010a)
**September 2009**	25	First fatal case in Germany (Robert Koch-Institute, 2009j)
	29	Authorization of first two pandemic vaccines (Focetria & Pandemrix) by European Commission (European Commission, 2009b)
**October 2009**	6	Authorization of third pandemic vaccine by EC (European Commission, 2009c)
	12	RKI: STIKO recommendations on priority groups for vaccination were published (Robert Koch-Institute, 2009c)
	14	Central information homepage on H1N1 was launched (Bundesministerium für Gesundheit, 2009)
	15	Number of reported pandemic A/H1N1 deaths: 2 (European Centre for Disease Prevention and Control, 2009ae)
	26	Germany began vaccination (Bundesministerium für Gesundheit, 2009)
**November 2009**	9	Notification regulation for suspected cases changed from week 46 onwards: Only A/H1N1 related deaths had to be reported. (Robert Koch-Institute, 2010a)

	11	Germany: 1st Impfgipfel at the Ministry of Health (Martin, 2010)
	16	Number of reported pandemic A/H1N1 deaths: 16 (European Centre for Disease Prevention and Control, 2009ah)
	Week 47	Vaccination coverage in persons ≥ 14 years: 4,6% (N=1000) (Walter et al.., 2011; Walter, Böhmer, Reiter, Krause, & Wichmann, 2012)
		Survey result: 18% (N=1000) perceived risk due to swine flu as great or partially great; 34% stated their perception of risk was low (Walter et al.., 2012)
	Week 48	Survey result: 69% (N=1001) believed it was not likely at all or rather unlikely that they would personally catch the A/H1N1influenza; 62% stated it was not likely at all or not likely that they would get vaccinated against pandemic A(H1N1).<br>80% (N=1001) perceived Health professionals to be the most trusted source of information (The Gallup Organization, 2010)
	Week 49	Vaccination coverage in persons ≥ 14 years: 6% (Walter et al.., 2011)
		Week 49: Pandemic Influenza A/H1N1 Surveillance in hospitals (PIKS) started (Buda et al., 2010)
**December 2009**	4	RKI stopped publishing daily reports (Robert Koch-Institute, 2009b)
	7	2nd Impfgipfel at the Ministry of Health (Martin, 2010)
	11	Number of reported pandemic A/H1N1 deaths: 94 (European Centre for Disease Prevention and Control, 2009ak)
	Week 51	Vaccination coverage in persons ≥ 14 years: 8% (Walter et al.., 2011)
		Survey result: 10% (1000) perceived risk due to swine flu as

		great or partially great (Walter et al.., 2012)
**January 2010**	Week 2	Survey result: 10% (N=1000) perceived risk due to swine flu as great or partially great (Walter et al.., 2012)
	15	Number of reported pandemic A/H1N1 deaths: 176 (European Centre for Disease Prevention and Control, 2010c)
**February 2010**	19	Number of reported pandemic A/H1N1 deaths: 235 (European Centre for Disease Prevention and Control, 2010d)
**March 2010**	Week 10	Survey result: 65% (N=4.005) stated their perception of risk was low (Walter et al.., 2012)
	19	Number of reported pandemic A/H1N1 deaths: 253 (Buda S, Influenza-Wochenbericht CW15 (2010)(European Centre for Disease Prevention and Control, 2010e)
**April 2010**	1	Pandemic Influenza A/H1N1 Surveillance in hospitals (PIKS) was terminated (Buda et al., 2010)
	26	Total number of confirmed cases: 225.729<br>Total number of deaths: 250<br>Hospitalizations: 7.882 (Buda et al., 2010)
**August 2010**	Aug	Vaccination coverage:<br>General population 8%<br>Healthcare workers 16%<br>Chronic disease (<65) 12%<br>Pregnant women 9% (Mereckiene et al.., 2012)
	10	WHO Director-General: World is no longer in a pandemic (World Health Organization, 2010c)

Chronology of key events during the 2009 A/H1N1 pandemic in Spain

Month	Day	Event
**April 2009**	4	Outbreak of influenza-like illness started in Veracruz, Mexico (European Centre for Disease Prevention and Control, 2010a)
	15	Novel Influenza A/H1N1 identified and isolated in USA (Centers for Disease Control and Prevention, 2009)
	24	WHO confirmed that the outbreak in Mexico was caused by a novel influenza virus (World Health Organization, 2009b)
		The Coordinating Centre for Health Alerts and Emergencies (CCAES) at the Spanish Ministry of Health and Social Policy issued a warning to the surveillance network in its daily report (Surveillance Group for New Influenza A(H1N1) Virus Investigation and Control in Spain, 2009)
		Ministry of Health and Social Policy (MHSP) published information and advice for travelers on its website (Surveillance Group for New Influenza A(H1N1) Virus Investigation and Control in Spain, 2009)
	25	WHO declared the outbreak of influenza A/H1N1 in Mexico and the US as a Public Health Emergency of International Concern (PHEIC) under IHR (2005) (World Health Organization, 2009c)
		National Influenza Preparedness and Response Plan activated (Surveillance Group for New Influenza A(H1N1) Virus Investigation and Control in Spain, 2009)
		CCAES distributed a case definition and protocols for infection control and management of cases and contacts (Surveillance Group for New Influenza A(H1N1) Virus Investigation and Control in Spain, 2009)

		Surveillance and disease control at airports started, meeting all flights from affected areas until 16 June (Dávila Cornejo et al., 2010)
	26	First 3 cases under investigation (Surveillance Group for New Influenza A(H1N1) Virus Investigation and Control in Spain, 2009)
	27	MHSP recommended to avoid any non-essential travel to Mexico (Surveillance Group for New Influenza A(H1N1) Virus Investigation and Control in Spain, 2009)
		WHO declared influenza pandemic alert phase 4 (World Health Organization, 2009d)
		First meeting of the Surveillance Subcommittee (Altogether 31 meetings until March 22) (Sierra Moros et al., 2010)
		First laboratory confirmed case of A/H1N1 in Spain (European Centre for Disease Prevention and Control, 2009f)
		Exclusive supply of antivirals to hospitals (Ministerio de Sanidad, Politica Social e Igualdad, 2010b)
	28	The Surveillance Subcommittee agreed on a protocol on case and contact management: Antivirals were offered to all cases and contacts. Isolation of cases and contacts was recommended (Santa-Olalla Peralta, Cortes García, Martínez Sánchez, et al., 2010; Sierra Moros et al., 2010)
		First meeting of the Subcommittee on Vaccines and Antivirals (SVA) (Ministerio de Sanidad, Politica Social e Igualdad, 2010b)
	29	WHO declared influenza pandemic alert phase 5 (World Health Organization, 2009e)
		First case of secondary transmission (Surveillance Group for New Influenza A(H1N1) Virus Investigation and Control in Spain, 2009)

	30	The European Union agreed on a common case definition for the new pandemic virus (European Commission, 2009a)
**May 2009**	1	Regional Influenza laboratories started initial testing (Santa-Olalla Peralta, Cortes García, Martínez Sánchez, et al., 2010)
	7	The CSP (Comisión de Salud Pública) approved a new case definition based on the EU case definition (Surveillance Group for New Influenza A(H1N1) Virus Investigation and Control in Spain, 2009)
	11	First confirmed tertiary case.<br>Number of confirmed cases: 98. 76 had a history of travel to Mexico (Surveillance Group for New Influenza A(H1N1) Virus Investigation and Control in Spain, 2009)
	13	CSP agreed on purchasing vaccine for 40% of the population. Enough vaccine for 18,3 million people (Ministerio de Sanidad, Politica Social e Igualdad, 2010b)
	20	Case and contact management protocol updated: Antivirals will be given only to cases with severe disease, those with risk factors and contacts with risk factors. Isolation of cases should be maintained. No quarantine of contacts (Santa-Olalla Peralta, Cortes García, Martínez Sánchez, et al., 2010)
	22	First outbreak without travel history at the Military Academy of Engineering in Hoyo de Manzanares (Ministerio de Sanidad, Politica Social e Igualdad, 2010a)
**June 2009**	11	WHO raised the level of influenza pandemic alert from phase 5 to phase 6 (World Health Organization, 2009h)<br>WHO considered severity of pandemic to be moderate (World Health Organization, 2009i)

	26	The CSP approved a surveillance strategy based on 5 points:<br>surveillance of severe cases,<br>the influenza surveillance through SISS (Spanish Influenza Surveillance System),<br>the monitoring of cluster of cases with acute respiratory infection (confirmation of first cases only; case-based notification not required),<br>monitoring of influenza or acute respiratory disease from the primary care computerized database and<br>case-based monitoring of flu cases in the community.<br>No need of identification, monitoring or administration of prophylaxis to contacts. (Santa-Olalla Peralta, Cortes García, Martínez Sánchez, et al., 2010)
**July 2009**	1	First fatal case in Spain (European Centre for Disease Prevention and Control, 2010a)
	27	Mitigation phase began (Sierra Moros et al., 2010)
	28	Case-based monitoring of cases in the community was ceased (Ministerio de Sanidad, Politica Social e Igualdad, 2010a)
		SVA agreed on population groups for antiviral treatment: cases requiring hospitalization and those at risk of complications. (approved by CSP on 29. July) (Ministerio de Sanidad, Politica Social e Igualdad, 2010a)
**August 2009**	7	AEMPS released recommendations on the use of antivirals in children under 1 year old, pregnant and breastfeeding women and people with swallowing problems (Agencia Española de Medicamentos y Productos Sanitarios, 2009a)
	14	Information campaign "Gripe A. La prevención es la major

		medida" started (Ministerio de Sanidad y Politica Social, 2009a)
	31	Agreement on priority groups for vaccination (Ministerio de Sanidad, Politica Social e Igualdad, 2010b)
**September 2009**	9	CSP approved surveillance strategy update: Investigation of clusters of cases only in those cases deemed necessary to make a special intervention. (Ministerio de Sanidad, Politica Social e Igualdad, 2010a)
	10	Number of reported pandemic A/H1N1 deaths: 25 (European Centre for Disease Prevention and Control, 2009ac)
	29	Authorization of first two pandemic vaccines (Focetria & Pandemrix) by European Commission (European Commission, 2009b)
**October 2009**	1	Vaccine became available for use (Venice II)
	6	Authorization of third pandemic vaccine by EC (European Commission, 2009c)
	15	Number of reported pandemic A/H1N1 deaths: 43 (European Centre for Disease Prevention and Control, 2009ae)
	29	Regular supply of antiviral drugs in pharmacies permitted (Agencia Española de Medicamentos y Productos Sanitarios, 2009b)
**November 2009**	16	Vaccination campaign started (Ministerio de Sanidad y Politica Social, 2009l)
		Authorization of a new pandemic vaccine Panenza in Spain (Agencia Española de Medicamentos y Productos Sanitarios, 2009c)
		Number of reported pandemic A/H1N1 deaths: 88 (European Centre for Disease Prevention and Control, 2009ah)

	21	AEMPS: Official recommendations on vaccination published (Agencia Española de Medicamentos y Productos Sanitarios, 2009d)
	Wee k 48	Survey result: 49% (N=1003) believed it was not likely at all or rather unlikely that they would personally catch the A/H1N1influenza; 66% stated it was not likely at all or not likely that they would get vaccinated against pandemic A(H1N1).<br>86% (N=1003) perceived Health professionals to be the most trusted source of information (The Gallup Organization, 2010)
**December 2009**	4	Surveillance Subcommittee eased the monitoring of severe cases (Ministerio de Sanidad, Politica Social e Igualdad, 2010a)
	11	Number of reported pandemic A/H1N1 deaths: 169 (European Centre for Disease Prevention and Control, 2009aj)
**January 2010**	15	Number of reported pandemic A/H1N1 deaths: 271 (European Centre for Disease Prevention and Control, 2010b)
**February 2010**	1	Case-based monitoring of severe cases was suspended (Ministerio de Sanidad, Politica Social e Igualdad, 2010a)
**April 2010**	1	Weekly reporting of new hospitalized cases and case-based notification of fatal cases was stopped (Ministerio de Sanidad, Politica Social e Igualdad, 2010a)
		Total number of reported deaths: 348 (Ministerio de Sanidad, Politica Social e Igualdad, 2010a)
	15	End of vaccination campaign (Ministerio de Sanidad, Politica Social e Igualdad, 2010b)
**August 2010**	Aug	Vaccination coverage:<br>General population 27%<br>Healthcare workers 12%

		Chronic disease (<65) 24%<br>Pregnant women 9% (Mereckiene et al.., 2012)
	10	WHO Director-General: World is no longer in a pandemic (World Health Organization, 2010c)

Chronology of key events during the 2009 A/H1N1 pandemic in the UK

Month	Day	Event
**April 2009**	4	Outbreak of influenza-like illness started in Veracruz, Mexico (European Centre for Disease Prevention and Control, 2010a)
	15	Novel Influenza A/H1N1 identified and isolated in USA (Centers for Disease Control and Prevention, 2009)
	24	WHO confirmed that the outbreak in Mexico was caused by a novel influenza virus (World Health Organization, 2009b)
	25	WHO declared the outbreak of influenza A/H1N1 in Mexico and the US as a Public Health Emergency of International Concern (PHEIC) under IHR (2005) (World Health Organization, 2009c)
	27	WHO declared influenza pandemic alert phase 4 (World Health Organization, 2009d)
		First two laboratory confirmed cases of A/H1N1 in the UK (European Centre for Disease Prevention and Control, 2009f)
		Containment strategy (Health Protection Agency, 2009d)<br>Initially, meeting all direct flights from Mexico. Borders were not closed, no restrictions on international or domestic travel and public mass gatherings (Hine, 2010)
	29	WHO declared influenza pandemic alert phase 5 (World Health Organization, 2009e)
		Gordon Brown announced: stockpile of antivirals was to be increased from 33.5 million to 50 million doses (Hine, 2010)
		First confirmed case in England; first UK school closure (Hine, 2010)
	30	The European Union agreed on a common case definition for the new pandemic virus (European Commission, 2009a)
		Information campaign started on TV, radio and in print media.

		Swine Flu Information Line was set up (Hine, 2010)
**May 2009**	1	First case of secondary transmission in the UK and Germany (European Centre for Disease Prevention and Control, 2009i)
	2	HPA put in place regional Flu Response Centers (Health Protection Agency, 2010c)
	3	5 cases of in country transmissions in Germany, Spain and UK (European Centre for Disease Prevention and Control, 2009j)
	6	Ministers agreed that containment phase should continue (Hine, 2010)
	11	Ministers decided to procure enough pre-pandemic vaccine for 45% of the population without waiting for Phase 6 (Hine, 2010)
	15	British Foreign& Commonwealth Office stopped to advice against all but essential travel to Mexico.<br>Agreements for up to 90 million doses of pre-pandemic vaccines were signed (Hine, 2010)
	16	Survey result: Percentage of very or fairly worried about the possibility of catching pandemic A/H1N1: 16,6 % (N= 1173) (Rubin, Potts, & Michie, 2010)
	20	HPA recommended mass prophylaxis at schools were any pupils were affected should cease (Department of Health. Scientific Advisory Group for Emergencies (SAGE), 2009a)
	21	Ministers decided not to change the prophylaxis policy at schools as recommended by HPA (Hine, 2010)
	22	HPA stopped meeting flights from Mexico (Health Protection Agency, 2009a)
**June 2009**	1	Scottish Flu Response Center was established to relieve the pressure on NHS 24 (Hine, 2010)

	10	Ministers agreed on policy for “hot spots” (Health Protection Agency, 2010c; Hine, 2010)
	11	WHO raised the level of influenza pandemic alert from phase 5 to phase 6 (World Health Organization, 2009h)<br>WHO considered severity of pandemic to be moderate (World Health Organization, 2009i)
	13	Total number of cases reached 1000 (Hine, 2010)
	15	First death reported in Europe; in the UK (European Centre for Disease Prevention and Control, 2009t)
	16	Survey result: Percentage of very or fairly worried about the possibility of catching pandemic A/H1N1: 19,3 % (N= 1050) (Rubin et al.., 2010)
	17	Ministers agreed to procure vaccine for 100% of the population (Hine, 2010)<br>DH`s Joint Committee on Vaccination and Immunization (JCVI) first meeting: priority groups for vaccination were discussed (Final advice on 8 October) (Department of Health. Joint Committee on Vaccination and Immunisation, 2009a)
	26	Contracts were signed with GlaxoSmithKline and Baxter Healthcare: 132 million doses of H1N1 vaccine (2 doses for the whole UK population) (Hine, 2010)
**July 2009**	2	Mitigation strategy started (Health Protection Agency, 2009d)
	6	MHRA developed a web-based reporting system for use by public and healthcare professionals wanting to report adverse reactions to antivirals and when available to vaccines (Medicines and Healthcare products Regulatory Agency, 2009a)
	13	Besides a few small changes, SAGE endorsed the JCVI`S advice

		concerning the priority groups for vaccination (Department of Health. Scientific Advisory Group for Emergencies (SAGE), 2009b)
	15	Survey result: Percentage of very or fairly worried about the possibility of catching pandemic A/H1N1: 32,9 % (N=1050) (Rubin et al.., 2010)
	16	Ministers agreed that the priority groups identified by SAGE would be vaccinated (Hine, 2010)<br>Publication of planning assumptions calculated by SAGE. Key figures: nearly 19 million cases, 2,8 million people with complications, 370.000 people hospitalized, up to 65.000 deaths (Department of Health, 2009e)
	23	National Pandemic Flu Service went live in England (Department of Health, 2009f)
	29	Ministers bought 30 million doses of additional Pandemrix vaccine to make up any possible shortfall (Hine, 2010)
**August 2009**	7	JCVI discussed the vaccine strategy and priority groups once more (Department of Health. Joint Committee on Vaccination and Immunisation, 2009b)
	13	UK published priority groups for the vaccination program (Department of Health, 2009g)
**September 2009**	3	Planning assumptions revised: reduction in hospitalization rate from 2% to 1%, reduction of upper case fatality rate from 0,35% to 0,1% (Department of Health, 2009i)
	10	Number of reported pandemic A/H1N1 deaths: 76 (European Centre for Disease Prevention and Control, 2009ac)
	13	Survey result: Likelihood of pandemic vaccine uptake (N=5175):<br>Very likely: 31,7 %

		Fairly likely: 24,4 %<br>Not very likely: 19,4 %<br>Very unlikely: 20,8 %<br>Not sure: 3,7 % (Rubin et al.., 2010)
	29	Authorization of first two pandemic vaccines (Focetria & Pandemrix) by European Commission (European Commission, 2009b)
**October 2009**	1	Web based reporting system across England introduced to collect information on all laboratory confirmed cases admitted to NHS trusts (Health Protection Agency, 2010b)
	6	Authorization of third pandemic vaccine by EC (European Commission, 2009c)
	8	JCVI reconfirmed the priority groups for vaccination and advised on dosage of vaccine (Department of Health. Joint Committee on Vaccination and Immunisation, 2009c)
	12	SAGE discussed and agreed the JCVI recommendations (Department of Health. Scientific Advisory Group for Emergencies (SAGE), 2009c)
	14	Four health ministers agreed that vaccination program should start at the same time throughout the UK (Hine, 2010)
	15	Number of reported pandemic A/H1N1 deaths: 95 (European Centre for Disease Prevention and Control, 2009ae)
	21	UK begins vaccination: front-line healthcare workers and patients who fall into at-risk categories (Department of Health, 2009k)
	22	Planning assumptions revised: Reasonable worst case for the clinical attack rate was reduced from 30% to 12%. Reasonable worst case for further deaths was reduced from 19.000 to 1.000

		(Department of Health, 2009m)
**November 2009**	5	Medicines and Healthcare products Regulatory Agency published suspected adverse reaction analysis on pandemic vaccines (Medicines and Healthcare products Regulatory Agency, 2009b)
	16	Number of reported pandemic A/H1N1 deaths: 185 (European Centre for Disease Prevention and Control, 2009ah)
	19	Phase two of vaccination program announced: children over 6 months and under 5 years (Department of Health, 2009u)
	Week 48	Survey result: 49% (N=1000) believed it was not likely at all or rather unlikely that they would personally catch the A/H1N1influenza; 37% stated it was not likely at all or not likely that they would get vaccinated against pandemic A(H1N1).<br>91% (N=1000) perceived Health professionals to be the most trusted source of information (The Gallup Organization, 2010)
	30	SAGE heard from its modelers that the pandemic had now effectively peaked (Department of Health. Scientific Advisory Group for Emergencies (SAGE), 2009d)
**December 2009**	11	Number of reported pandemic A/H1N1 deaths: 283 (European Centre for Disease Prevention and Control, 2009aj)
	23	Department of Health wrote to Baxter Healthcare to stop supply of Celvapan® from 28 February (Hine, 2010)
**January 2010**	8	JCVI statement: vaccination of further groups of people is not recommended (Department of Health. Joint Committee on Vaccination and Immunisation, 2010)
	11	SAGE met for the last time (Department of Health. Scientific Advisory Group for Emergencies (SAGE), 2010)
	14	Agreement to start negotiating with GlaxoSmithKline (GSK) over

		ceasing the contract and suspending Pandemrix deliveries from 16 January (Hine, 2010)
	15	Number of reported pandemic A/H1N1 deaths: 362 (European Centre for Disease Prevention and Control, 2010b)
**February 2010**	4	Agreement that vaccination program was not extended to other healthy age groups. Strategic reserve of 15 million doses was set up (Hine, 2010)
	11	National Pandemic Flu Service was closed down (Hine, 2010)
**March 2010**	12	Reported deaths across the UK: 440 (Pebody et al., 2010)
	18	H1N1 (2009) swine flu vaccine provided for protection of travelers to Southern Hemisphere countries (Department of Health, 2010c)
**April 2010**	1	Antivirals were no longer available from national stockpiles; Swine Flu Information Line was closed down; Treatment of cases returned to business as usual (Hine, 2010)
	6	Agreement with GSK to only take deliveries of just under 35 million doses of Pandemrix® (The Secretary of State for Health, 2010)
	15	Total number of deaths: 474 (Department of Health, 2010d)
**August 2010**	Aug	Vaccination coverage<br>**England:**<br>Chronic disease (<65) 37,6%, including pregnant women<br>Children (6 month to 5) 23,6%<br>Healthcare workers 40,3%<br>Vaccine uptake in Wales was similar.<br>**Northern Ireland:**<br>Chronic disease (<65) 86,5%<br>Children 38,3%<br>Healthcare workers 47,7%

		**Scotland:**<br>Chronic disease (<65) 54,5%<br>Children 44,6%<br>Healthcare workers 55,1%<br>(Health Protection Agency, 2010b)
	10	WHO Director-General: World is no longer in a pandemic (World Health Organization, 2010c)

References

Agencia Española de Medicamentos y Productos Sanitarios. (2009a, August 7). Nota informativa para profesionales sanitarios. Actualización de la nota informativa sobre la preparación y administración de oseltamivir y zanamivir en ninos menores de 1 ano, gestantes y mujeres en periodo de lactancia, y personas con problemas de deglución. Available from: http://www.aemps.gob.es/informa/notasInformativas/medicamentosUsoHumano/2009/docs/NI-Oseltamivir-Zanamivir_agosto-2009.pdf

Agencia Española de Medicamentos y Productos Sanitarios. (2009b, November 4). Informe mensual sobre medicamentos de uso humano y productos sanitarios.Octubre 2009. Available from: http://www.aemps.gob.es/informa/informeMensual/2009/octubre/docs/informe-mensual_octubre-2009.pdf

Agencia Española de Medicamentos y Productos Sanitarios. (2009c, November 16). Nota inormativa para profesionales sanitarios. Autorización de una nueva vacuna frente al virus de la gripe A (H1N1) pandémico en Espana. Available from: http://www.aemps.gob.es/informa/notasInformativas/medicamentosUsoHumano/vacunas/2009/NI_autorizacion-vacuna-gripe-A.htm

Agencia Española de Medicamentos y Productos Sanitarios. (2009d, Dezember 21). Campana de vacunación frente al nuevo virus gripal pandémico H1N1. Recomendaciones oficiales. Available from: http://www.aemps.gob.es/informa/notasInformativas/medicamentosUsoHumano/vacunas/2009/docs/NI_campana-vacunacion-H1N1_recomendaciones-oficiales.pdf

Agüero, F., Adell, M. N., Giménez, A. P., Medina, M. J. L., & Continente, X. G. (2011). Adoption of preventive measures during and after the 2009 influenza A (H1N1) virus pandemic peak in Spain. *Preventive Medicine*, *53*, 203–206.

Amato-Gauci, A., Zucs, P., Snacken, R., Ciancio, B., Lopez, V., Broberg, E., … on behalf of the European Influenza Surveillance Network (EISN). (2010). Surveillance trends of the 2009 influenza A(H1N1) pandemic in Europe. *Euro Surveillance*, *16*(26).

Available from: http://www.eurosurveillance.org/ViewArticle.aspx?ArticleId=19903

Andersen, P. H. (2009a). INFLUENZA A H1N1 OF NEW SUBTYPE (SWINE INFLUENZA). *EPI-News. National Surveillance of Communicable Diseases*, (18). Available from: http://www.ssi.dk/English/News/EPI-NEWS/~/media/Indhold/EN%20-%20engelsk/EPI-NEWS/2009/pdf/EPI-NEWS%20-%202009%20-%20No%2018.ashx

Andersen, P. H. (2009b). NOVEL INFLUENZA A (H1N1) –CLARIFICATION OF GUIDELINES. *EPI-News. National Surveillance of Communicable Diseases*, (21). Available from: http://www.ssi.dk/English/News/EPI-NEWS/~/media/Indhold/EN%20-%20engelsk/EPI-NEWS/2009/pdf/EPI-NEWS%20-%202009%20-%20No%2021.ashx

Andersen, P. H. (2009c). INFLUENZA A (H1N1)v – CLARIFICATION OF NEW GUIDELINES. *EPI-News. National Surveillance of Communicable Diseases*, (27-29). Available from: http://www.ssi.dk/English/News/EPI-NEWS/~/media/Indhold/EN%20-%20engelsk/EPI-NEWS/2009/pdf/EPI-NEWS%20-%202009%20-%20No%2027-29.ashx

Andersen, P. H. (2009d). FAQs ON PANDEMIC VACCINE (PANDEMRIX®). *EPI-News. National Surveillance of Communicable Diseases*, (43). Available from: http://www.ssi.dk/English/News/EPI-NEWS/~/media/Indhold/EN%20-%20engelsk/EPI-NEWS/2009/pdf/EPI-NEWS%20-%202009%20-%20No%2043.ashx

Andersen, P. H. (2009e). INFLUENZA EPIDEMIC. *EPI-News. National Surveillance of Communicable Diseases*, (46). Available from: http://www.ssi.dk/English/News/EPI-NEWS/~/media/Indhold/EN%20-%20engelsk/EPI-NEWS/2009/pdf/EPI-NEWS%20-%202009%20-%20No%2046.ashx

Andersen, P. H. (2009f). INFLUENZA EPIDEMIC UPDATE. *EPI-News. National Surveillance of Communicable Diseases*, (49). Available from: http://www.ssi.dk/English/News/EPI-NEWS/~/media/Indhold/EN%20-%20engelsk/EPI-NEWS/2009/pdf/EPI-NEWS%20-%202009%20-%20No%2049.ashx

Andersen, P. H. (2010a). Influenza season 2009-2010. *EPI-News. National Surveillance of Communicable Diseases*, (23). Available from: http://www.ssi.dk/English/News/EPI-NEWS/2010/No%2023%20-%202010.aspx

Andersen, P. H. (2010b). The influenza pandemic. *EPI-News. National Surveillance of Communicable Diseases*, (1). Available from: http://www.ssi.dk/English/News/EPI-NEWS/2010/No%201%20-%202010.aspx

Aramaki, E., Maskawa, S., Morita, M. (2011): Twitter Catches The Flu: Detecting Influenza Epidemics using Twitter. Proceedings of the 2011 Conference on Empirical Methods in Natural Language Processing; 1568-1576. Available from: http://luululu.sakura.ne.jp/paper/2011/EMNLP.pdf (21.07.2014)

Buda, S., Köpke, K., Luchtenberg, M., Schweiger, B., Biere, B., Duwe, S., … Haas, W. (2010). Bericht zur Epidemiologie der Influenza in Deutschland Saison 2009/10. Robert Koch-Institute. Available from: http://influenza.rki.de/Saisonberichte/2009.pdf

Bundesministerium der Justiz. (2009). Bundesministerium für Gesundheit. Verordnung über die Meldepflicht bei Influenza, die durch das erstmals im April 2009 in Nordamerika aufgetretene neue Virus („Schweine-Grippe") hervorgerufen wird. Vom 30. April 2009. *Bundesanzeiger*, *61*(1), 1589.

Bundesministerium für Gesundheit. (2009, Oktober 14). Pressemitteilung: Informationsangebot des Bundesgesundheitsministeriums zum Start der Impfungen gegen die Neue Grippe. Available from: http://www.bmg.bund.de/fileadmin/redaktion/pdf_pressemeldungen/2009/091014-PM.pdf

Bundesministerium für Gesundheit, Bundeszentrale für gesundheitliche Aufklärung, Robert Koch-Institute, & Paul-Ehrlich-Institute. (2009a, Oktober). Impfung gegen die Neue Grippe („Schweinegrippe"). Information für Menschen mit chronischen Erkrankungen. Available from: http://www.thueringen.de/imperia/md/content/tmsfg/aktuell/h1n1/rz_final_chron.erkrankungen.pdf

Bundesministerium für Gesundheit, Bundeszentrale für gesundheitliche Aufklärung, Robert Koch-Institute, & Paul-Ehrlich-Institute. (2009b, Oktober). Impfung gegen die Neue Grippe („Schweinegrippe"). Information für medizinisches Personal in Krankenhäusern, Praxen und Laboratorien. Available from: http://www.cremlingen.de/content/files/downloads/merkblatt_med_pers.pdf

Bundesministerium für Gesundheit, Bundeszentrale für gesundheitliche Aufklärung, Robert Koch-Institute, & Paul-Ehrlich-Institute. (2009c, Oktober). Impfung gegen die Neue Grippe („Schweinegrippe"). Information für Schwangere. Available from: http://www.berlin.de/imperia/md/content/landesverwaltungsamt/beihilfe/formulareundmerkblaetter/mb_bm_ges_schweinegrippeinfo_fuer_schwangere.pdf?start&ts=1256892413&file=mb_bm_ges_schweinegrippeinfo_fuer_schwangere.pdf

Bundesministerium für Gesundheit, Bundeszentrale für gesundheitliche Aufklärung, Robert Koch-Institute, & Paul-Ehrlich-Institute. (2009d, Oktober). Impfung gegen die Neue Grippe („Schweinegrippe"). Information für Angehörige von Polizei und Feuerwehr. Available from: http://www.muenster.de/stadt/gesundheitsamt/pdf/neue-grippe_polizei-feuerwehr.pdf

Bundesministerium für Gesundheit, Bundeszentrale für gesundheitliche Aufklärung, Robert Koch-Institute, & Paul-Ehrlich-Institute. (2009e, Oktober 1). Impfung gegen die Neue Grippe („ Schweinegrippe"). Available from: http://www.kreis-offenbach.de/PDF/Impfung_gegen_die_Neue_Grippe_Schweinegrippe_Merkblatt.PDF?ObjSvrID=350&ObjID=4690&ObjLa=1&Ext=PDF&WTR=1&_ts=1339506385

Bundeszentrale für gesundheitliche Aufklärung. (2009a, Juli 30). Schweinegrippe. Empfehlungen zum Verhalten im Verdachts- und Krankheitsfall. Available from: http://www.bzga.de/presse/pressearchiv/?jahr=2009&nummer=538

Bundeszentrale für gesundheitliche Aufklärung. (2009b, August 5). Influenza A/H1N1: Hygiene- und Verhaltenstipps im Urlaub. Available from: http://www.bzga.de/presse/pressearchiv/?jahr=2009&nummer=540

Bundeszentrale für gesundheitliche Aufklärung. (2009c, Oktober 22). Bundeszentrale für gesundheitliche Aufklärung startet Schulaktion zum richtigen Hygieneverhalten. Available from: http://www.bzga.de/presse/pressearchiv/?jahr=2009&nummer=551

Bush, R. M., Budowle (Ed), B., Schutzer (Ed), S. E., Breeze (Ed), R. G., Keim (Ed), P. S., & Morse (Ed), S. A. (2011). Influenza Forensics. In *Microbiol Forensics* (2. Aufl., S. 109–135). Elsevier.

Centers for Disease Control and Prevention. (2009). Swine Influenza A (H1N1) Infection in Two Children -Southern California, March-April 2009. *Morbidity and Mortality Weekly Report*, *58*, 1–3.

Centro Nacional de Epidemiología. Instituto de Salud Carlos III. (2009, Juli 1). Informe semanal del Sistema de Vigilancia de la Gripe en España (SVGE). Available from: http://vgripe.isciii.es/gripe/inicio.do;jsessionid=17C592C4767107866677EA4B0CF47BF9

Centro Nacional de Epidemiología. Instituto de Salud Carlos III. (n.y.). Vigilancia de la gripe en Espana. Evolución de la gripe pandémica por AnH1N1. (Desde la semana 20/2009 hasta la semana 20/2010). Available from:

http://www.isciii.es/ISCIII/es/contenidos/fd-servicios-cientifico-tecnicos/fd-vigilancias-alertas/fd-enfermedad-es/Vigilancia_de_la_gripe_en_Espana_Evolucion_de_la_pandemia_por_AnH1N1_Temporada_2009-2010.pdf (20.06.2014)

Dacey, G., Blake, A., Morgan-Lewis, L., Riley, P., Simpson, J., Barratt, J., … Harrison, C. (2010). Assessment Report on the EU-wide Response to Pandemic (H1N1) 2009.Covering the period 24 April 2009 – 31 August 2009 (excluding vaccine policy issues). Available from: http://ec.europa.eu/health/communicable_diseases/docs/assessment_response_en.pdf

Dávila Cornejo, M., Aramburu Celigueta, C., Morte Esteban, S., Gil, I. V., Iglesiai Garcia, J., & Gonzáles Gutiérrez-Solana, O. (2010). Health Control at International Borders. The role of Foreign Health during The Containment Phases of the Pandemic (H1N1) 2009. *Revista Española de Salud Pública*, *84*(5), 507–516.

Department of Health. (2009a, April 30). Important Information about swine flu. Available from: http://www.dh.gov.uk/prod_consum_dh/groups/dh_digitalassets/@dh/@en/documents/digitalasset/dh_098680.pdf

Department of Health. (2009b, Juli 2). Swine Flu Pandemic: From Containment to Treatment. Guidance for the NHS. Available from:http://www.dh.gov.uk/prod_consum_dh/groups/dh_digitalassets/documents/digitalasset/dh_102021.pdf

Department of Health. (2009c, Juli 2). Swine Flu: From Containment to Treatment. Available from: http://www.dh.gov.uk/prod_consum_dh/groups/dh_digitalassets/@dh/@en/documents/digitalasset/dh_101955.pdf

Department of Health. (2009d, Juli 2). Swine Flu: From Containment to Treatment. Scientific Issues. Available from: http://www.dh.gov.uk/prod_consum_dh/groups/dh_digitalassets/@dh/@en/documents/digitalasset/dh_101988.pdf

Department of Health. (2009e, Juli 16). Swine Flu. UK Planning Assumptions. Available from:

http://www.dh.gov.uk/prod_consum_dh/groups/dh_digitalassets/documents/digitalasset/dh_102891.pdf

Department of Health. (2009f, Juli 23). A (H1N1) Swine Influenza: National Pandemic Flu Service launches today. Available from: http://www.dh.gov.uk/prod_consum_dh/groups/dh_digitalassets/documents/digitalasset/dh_103229.pdf

Department of Health. (2009g, August 13). Priority groups for the vaccination programme. Available from: http://webarchive.nationalarchives.gov.uk/+/www.dh.gov.uk/en/Publichealth/Flu/Swineflu/InformationandGuidance/Vaccinationprogramme/DH_105455

Department of Health. (2009h, September). Seasonal Flu. Why you should have the vaccination. Available from: http://www.dh.gov.uk/prod_consum_dh/groups/dh_digitalassets/documents/digitalasset/dh_105356.pdf

Department of Health. (2009i, September 3). Swine Flu. UK Planning Assumptions. Available from: http://www.dh.gov.uk/prod_consum_dh/groups/dh_digitalassets/documents/digitalasset/dh_104843.pdf

Department of Health. (2009j, September 29). Pandemic influenza. Recommendations on the use of antiviral medicines for pregnant women, women who are breastfeeding and children under the age of one year. Available from: http://www.dh.gov.uk/prod_consum_dh/groups/dh_digitalassets/documents/digitalasset/dh_106148.pdf

Department of Health. (2009k, Oktober 15). The H1N1 swine flu vaccination programme 2009-2010. Available from: http://www.dh.gov.uk/prod_consum_dh/groups/dh_digitalassets/@dh/@en/documents/digitalasset/dh_107190.pdf

Department of Health. (2009l, Oktober 15). Antiviral prophylaxis. Guidance on the use of prophylaxis with antiviral medicines during the H1N1 (swine flu) pandemic. Available from: http://www.dh.gov.uk/prod_consum_dh/groups/dh_digitalassets/documents/digitalasset/dh_107132.pdf

ECOM

Department of Health. (2009m, Oktober 22). Swine Flu. Guidance for planners. Available from: http://www.dh.gov.uk/prod_consum_dh/groups/dh_digitalassets/@dh/@en/@ps/@sta/@perf/documents/digitalasset/dh_107428.pdf

Department of Health. (2009n, Oktober 22). Swine Flu Vaccination: what you need to know. Available from: http://www.dh.gov.uk/prod_consum_dh/groups/dh_digitalassets/@dh/@en/@ps/@sta/@perf/documents/digitalasset/dh_109109.pdf

Department of Health. (2009o, Oktober 28). Health and Social Care Workers and Pandemic Influenza. Information for staff who are pregnant or in other at-risk groups. Available from: http://www.dh.gov.uk/prod_consum_dh/groups/dh_digitalassets/documents/digitalasset/dh_108365.pdf

Department of Health. (2009p, Oktober 28). Clinical Professionals Brief on Swine Flu Vaccination. Available from: http://www.dh.gov.uk/prod_consum_dh/groups/dh_digitalassets/documents/digitalasset/dh_107651.pdf

Department of Health. (2009q, Oktober 30). Pandemic H1N1 2009 Influenza: Clinical Management Guidelines for Adults and Children. Available from: http://www.dh.gov.uk/prod_consum_dh/groups/dh_digitalassets/@dh/@en/@ps/@sta/@perf/documents/digitalasset/dh_110617.pdf

Department of Health. (2009r, November 11). Swine Flu and Pregnancy. How to protect yourself and your baby. Available from: http://www.dh.gov.uk/prod_consum_dh/groups/dh_digitalassets/@dh/@en/documents/digitalasset/dh_108154.pdf

Department of Health. (2009s, November 12). Swine Flu. If you can´t catch it, you can´t pass it on. Available from: http://www.dh.gov.uk/prod_consum_dh/groups/dh_digitalassets/@dh/@en/@ps/@sta/@perf/documents/digitalasset/dh_108392.pdf

Department of Health. (2009t, November 19). Swine Flu Vaccine Deliveries and Distribution - Frequently Asked Questions. Available from: http://www.dh.gov.uk/prod_consum_dh/groups/dh_digitalassets/@dh/@en/@ps/@sta/@perf/documents/digitalasset/dh_108835.pdf

Department of Health. (2009u, November 20). Extending the H1N1 swine flu vaccination programme 2009/2010. Available from: http://www.dh.gov.uk/prod_consum_dh/groups/dh_digitalassets/@dh/@en/documents/digitalasset/dh_108896.pdf

Department of Health. (2009v, Dezember 8). A (H1N1) swine flu influenza: phase two of the vaccination programme; children over 6 months and under 5 years. Available from: http://www.dh.gov.uk/prod_consum_dh/groups/dh_digitalassets/@dh/@en/documents/digitalasset/dh_109825.pdf

Department of Health. (2009w, Dezember 15). Swine Flu Vaccination: information for parents of children over six months and under five years old. Available from: http://www.direct.gov.uk/prod_consum_dg/groups/dg_digitalassets/@dg/@en/documents/digitalasset/dg_183752.pdf

Department of Health. (2010a). Pandemic influenza preparedness programme: Statistical Legacy Group - a report for the Chief Medical Officer. Available from: http://www.dh.gov.uk/prod_consum_dh/groups/dh_digitalassets/@dh/@en/@ps/documents/digitalasset/dh_122754.pdf

Department of Health. (2010b, Januar). Swine Flu. Information Sheet for asylum seekers, refugees and other foreign nationals in the UK. Available from: http://www.dh.gov.uk/en/Publicationsandstatistics/Publications/PublicationsPolicyAndGuidance/DH_110808

Department of Health. (2010c, März 18). Pandemic H1N1 (2009) swine flu vaccines for travel use. Available from: http://www.dh.gov.uk/prod_consum_dh/groups/dh_digitalassets/documents/digitalasset/dh_114372.pdf

Department of Health. (2010d, April 15). Pandemic H1N1 (2009) Influenza: Chief Medical Officer’s Statistical Update. Available from: http://www.dh.gov.uk/prod_consum_dh/groups/dh_digitalassets/documents/digitalasset/dh_115427.pdf

Department of Health, & Royal College of Obstetricians and Gynaecologists. (2009, Oktober 30). Pandemic H1N1 2009 Influenza: Clinical Management Guidelines for Pregnancy. Available from: http://www.dh.gov.uk/prod_consum_dh/groups/dh_digitalassets/@dh/@en/@ps/@sta/@perf/documents/digitalasset/dh_110618.pdf

Department of Health. Joint Committee on Vaccination and Immunisation. (2009a, Juni 17). Draft minutes of the meeting held on 17 June 2009. Available from: http://www.dh.gov.uk/prod_consum_dh/groups/dh_digitalassets/@dh/@ab/documents/digitalasset/dh_116040.pdf

Department of Health. Joint Committee on Vaccination and Immunisation. (2009b, August 7). Minute of the meeting held on 7 August 2009. Available from: http://www.dh.gov.uk/prod_consum_dh/groups/dh_digitalassets/@dh/@ab/documents/digitalasset/dh_108037.pdf

Department of Health. Joint Committee on Vaccination and Immunisation. (2009c, Oktober 8). Minute of the meeting held on 8 October 2009. Available from: http://www.dh.gov.uk/prod_consum_dh/groups/dh_digitalassets/@dh/@ab/documents/digitalasset/dh_108833.pdf

Department of Health. Joint Committee on Vaccination and Immunisation. (2010, Januar 8). Advice on the H1N1v vaccination programme Friday 8th January 2010. Available from: http://www.dh.gov.uk/prod_consum_dh/groups/dh_digitalassets/@dh/@ab/documents/digitalasset/dh_112665.pdf

Department of Health. Scientific Advisory Group for Emergencies (SAGE). (2009a, Mai 20). Swine Flu. Minutes of a Meeting held in the Boardroom, Richmond House, 79 Whitehall, London SW1A 2NS, Department of Health at 10.00 am on 20 May 2009. Available from: http://www.dh.gov.uk/prod_consum_dh/groups/dh_digitalassets/@dh/@ab/documents/digitalasset/dh_126077.pdf

Department of Health. Scientific Advisory Group for Emergencies (SAGE). (2009b, Juli 13). Swine Flu. Minutes of a Meeting held in the Boardroom, Richmond House, 79 Whitehall, London, SW1A 2NS at 11.00 am on 13th July 2009. Available from: http://www.dh.gov.uk/prod_consum_dh/groups/dh_digitalassets/@dh/@ab/documents/digitalasset/dh_126063.pdf

Department of Health. Scientific Advisory Group for Emergencies (SAGE). (2009c, Oktober 12). Swine Flu. Minutes of a Meeting held in 35 Great Smith, London, SW1P 3PQ at 10.30 am on 12th October 2009. Available from: http://www.dh.gov.uk/prod_consum_dh/groups/dh_digitalassets/@dh/@ab/documents/digitalasset/dh_126069.pdf

Department of Health. Scientific Advisory Group for Emergencies (SAGE). (2009d, November 30). Swine Flu. Minutes of a Meeting held in Avonmouth House, 6 Avonmouth Street, London, SE1 6NX at 2.30 pm on 30th November 2009. Available from: http://www.dh.gov.uk/prod_consum_dh/groups/dh_digitalassets/@dh/@ab/documents/digitalasset/dh_126071.pdf

Department of Health. Scientific Advisory Group for Emergencies (SAGE). (2010, Januar 11). Swine Flu. Minutes of a Meeting held in the Boardroom, Richmond House, 79 Whitehall, London, SWIA 2NS at 10.30 am on 11th January 2010. Available from: http://www.dh.gov.uk/prod_consum_dh/groups/dh_digitalassets/@dh/@ab/documents/digitalasset/dh_126072.pdf

Die Beauftragte der Bundesregierung für Migration, Flüchtlinge und Integration. (2009, Juli 15). Tipps und Informationen zur Neuen Grippe A/H1N1. Available from: http://www.bundesregierung.de/Content/DE/Artikel/IB/Artikel/Themen/Gesellschaft/Gesundheit/2009-07-15-neue-grippe.html

Donaldson, L. ., Rutter, P. ., Ellis, B. ., Greaves, F. E. ., Mytton, O. ., Pebody, R. G., & Yardley, I. . (2009). Mortality from pandemic A/H1N1 2009 influenza in England: public health surveillance study. *BMJ*, *339:b5213*.

Ecom@EU Study Group. (2011). Effective Communication in Outbreak Management: development of an evidence-based tool for Europe (Ecom@EU). Proposal.

European Centre for Disease Prevention and Control. (2009a). ECDC Technical Report. Guide to public health measures to reduce the impact of influenza pandemics in Europe: 'The ECDC Menu'. Available from: http://www.ecdc.europa.eu/en/publications/Publications/0906_TER_Public_Health_Measures_for_Influenza_Pandemics.pdf

European Centre for Disease Prevention and Control. (2009b). Archive: Q&A for the general public on vaccines and vaccination in relation to the 2009 influenza A(H1N1) pandemic. Available from: http://www.ecdc.europa.eu/en/healthtopics/pandemic_preparedness/2009_pandemic_vaccines/Pages/QA_gp_pandemic_vaccines.aspx

European Centre for Disease Prevention and Control. (2009c). Archive: Q&A for health professionals on vaccines and vaccination in relation to the 2009 influenza A(H1N1) pandemic. Available from:

http://www.ecdc.europa.eu/en/healthtopics/pandemic_preparedness/2009_pandemic_vaccines/Pages/QA_hp_pandemic_vaccines.aspx

European Centre for Disease Prevention and Control. (2009d, April 24). ECDC Threat Assessment - UPDATE Human cases of swine influenza without apparent exposure to pigs, United States and Mexico 24 April 2009. Available from: http://ecdc.europa.eu/en/publications/Publications/090424_TER_Influenza_AH1N1_TA_Swine_influenza_US-Mexico.pdf

European Centre for Disease Prevention and Control. (2009e, April 25). Situation Report – Swine Influenza Mexico/United States. Available from: http://ecdc.europa.eu/en/healthtopics/Documents/090425_InfluenzaAH1N1_Situation_Report_0800hrs.pdf

European Centre for Disease Prevention and Control. (2009f, April 28). Situation Report – Infections of novel flu virus (A/H1N1). Available from: http://ecdc.europa.eu/en/healthtopics/Documents/090428_InfluenzaAH1N1_Situation_Report_0800hrs.pdf

European Centre for Disease Prevention and Control. (2009g, April 30). ECDC Threat Assessment.Public health issue Implication for Europe of the identification in North America of human cases of influenza A/H1N1, with a unique gene segment combination. Available from: http://ecdc.europa.eu/en/healthtopics/H1N1/Documents/1001_RA_090430.pdf

European Centre for Disease Prevention and Control. (2009h, April 30). ECDC Situation Report. Infections of novel influenza virus A(H1N1). Available from: http://ecdc.europa.eu/en/healthtopics/Documents/090430_InfluenzaAH1N1_Situation_Report_0800hrs.pdf

European Centre for Disease Prevention and Control. (2009i, Mai 2). ECDC Situation Report. Influenza A(H1N1) infection. Available from: http://ecdc.europa.eu/en/healthtopics/Documents/090502_InfluenzaAH1N1_Situation_Report_0800hrs.pdf

European Centre for Disease Prevention and Control. (2009j, Mai 3). ECDC Situation Report. Influenza A(H1N1) infection. Available from: http://ecdc.europa.eu/en/healthtopics/Documents/090503_InfluenzaAH1N1_Situation_Report_1030hrs.pdf

European Centre for Disease Prevention and Control. (2009k, Mai 4). ECDC Health Information. Influenza A(H1N1) virus:how to protect yourself. Available from: http://www.ecdc.europa.eu/en/healthtopics/Documents/0905_Influenza_A%28H1N1%29_how_to_protect_yourself.pdf

European Centre for Disease Prevention and Control. (2009l, Mai 12). ECDC Situation Report. Influenza A(H1N1) infection. Available from: http://ecdc.europa.eu/en/healthtopics/Documents/090512_InfluenzaAH1N1_Situation_Report_0800hrs.pdf

European Centre for Disease Prevention and Control. (2009m, Mai 18). ECDC Information for Travellers. Influenza A(H1N1). Available from: http://www.ecdc.europa.eu/en/healthtopics/Documents/0905_Influenza_AH1N1_Info_for_Travellers.pdf

European Centre for Disease Prevention and Control. (2009n, Mai 19). ECDC Health Information. Personal protective measures for reducing the risk of acquiring or transmitting human influenza. Available from: http://ecdc.europa.eu/en/healthtopics/Documents/09_07_personal_protective_measures_ECDC-2009-0001-00-00-ENEN_final.pdf

European Centre for Disease Prevention and Control. (2009o, Mai 19). ECDC Interim Guidance. Interim ECDC public health guidance on case and contact management for the new influenza A(H1N1) virus infection. Available from: http://www.ecdc.europa.eu/en/publications/Publications/0905_GUI_Influenza_AH1N1_Public_Health_Guidance_on_Case_and_Contact_Management.pdf

European Centre for Disease Prevention and Control. (2009p, Mai 20). ECDC Risk Assessment. Human cases of influenza A(H1N1). Available from: http://ecdc.europa.eu/en/healthtopics/H1N1/Documents/1001_RA_090520.pdf

European Centre for Disease Prevention and Control. (2009q, Mai 25). ECDC Situation Report. Influenza A(H1N1) infection. Available from: http://ecdc.europa.eu/en/healthtopics/Documents/090525_InfluenzaAH1N1_Situation_Report_1700hrs.pdf

European Centre for Disease Prevention and Control. (2009r, Juni 6). ECDC Interim Guidance. Mitigation and delaying (or 'containment')strategies as the new influenza A(H1N1) virus comes into Europe. Available from: http://ecdc.europa.eu/en/publications/publications/0906_gui_influenza_ah1n1_mitigation_and_delaying_strategies_for_the_influenza_in_europe.pdf

ECOM

European Centre for Disease Prevention and Control. (2009s, Juni 12). ECDC Interim Risk Assessment. Human cases of influenza A(H1N1)v. Available from: http://ecdc.europa.eu/en/healthtopics/H1N1/Documents/1001_RA_090612.pdf

European Centre for Disease Prevention and Control. (2009t, Juni 15). ECDC Situation Report. Influenza A(H1N1)v infection. Available from: http://ecdc.europa.eu/en/healthtopics/Documents/090615_Influenza_AH1N1_Situation_Report_1700hrs.pdf

European Centre for Disease Prevention and Control. (2009u, Juli 1). ECDC Threat Assessment. First isolation of a secondary oseltamivir-resitant A(H1N1)v strain in Denmark. Available from: http://ecdc.europa.eu/en/healthtopics/Documents/0906_Influenza_AH1N1_ECDC_Threat_Assessment_First_isolation_of_a_secondary_oseltamivir_resistant_strain_in_Denmark.pdf

European Centre for Disease Prevention and Control. (2009v, Juli 2). ECDC Situation Report. Influenza A(H1N1)v infection. Available from: http://ecdc.europa.eu/en/healthtopics/Documents/090702_Influenza_AH1N1_Situation_Report_1700hrs.pdf

European Centre for Disease Prevention and Control. (2009w, Juli 20). ECDC Interim Risk Assessment. Influenza A(H1N1) 2009 pandemic. Available from: http://ecdc.europa.eu/en/healthtopics/H1N1/Documents/1001_RA_090720.pdf

European Centre for Disease Prevention and Control. (2009x, Juli 20). Managing schools during the current pandemic (H1N1) 2009 – Reactive and proactive school closures in Europe. Available from: http://www.ecdc.europa.eu/en/activities/sciadvice/Lists/ECDC%20Reviews/ECDC_DispForm.aspx?List=512ff74f-77d4-4ad8-b6d6-bf0f23083f30&ID=631

European Centre for Disease Prevention and Control. (2009y, August). ECDC Health Education. On public health use of influenza antivirals during influenza pandemics (with particular reference to the pandemic (H1N1) 2009). Available from: http://www.ecdc.europa.eu/en/healthtopics/Documents/0908_Influenza_AH1N1_On_Public_Health_Use_of_Influenza_Antivirals_during_Influenza_Pandemics.pdf

European Centre for Disease Prevention and Control. (2009z, August). ECDC Interim Guidance. Use of specific pandemic influenza vaccines during the H1N1 2009 pandemic. Available from:

http://www.ecdc.europa.eu/en/publications/Publications/0908_GUI_Pandemic_Influenza_Vaccines_during_the_H1N1_2009_Pandemic.pdf

European Centre for Disease Prevention and Control. (2009aa, August 4). ECDC Situation Report. Pandemic influenza (H1N1) 2009. Available from: http://ecdc.europa.eu/en/healthtopics/Documents/090804_Influenza_AH1N1_Situation_Report_1700hrs.pdf

European Centre for Disease Prevention and Control. (2009ab, August 21). ECDC Interim Risk Assessment. Pandemic (H1N1) 2009 influenza. Available from: http://ecdc.europa.eu/en/healthtopics/H1N1/Documents/1001_RA_090821.pdf

European Centre for Disease Prevention and Control. (2009ac, September 15). ECDC Daily Update. Pandemic (H1N1) 2009. Available from: http://www.ecdc.europa.eu/en/healthtopics/Documents/090915_Influenza_AH1N1_Situation_Report_1700hrs.pdf

European Centre for Disease Prevention and Control. (2009ad, September 25). ECDC Interim Risk Assessment. Pandemic H1N1 2009. Available from: http://ecdc.europa.eu/en/healthtopics/H1N1/Documents/1001_RA_090925.pdf

European Centre for Disease Prevention and Control. (2009ae, Oktober 15). ECDC Daily Update. Pandemic (H1N1) 2009. Available from: http://www.ecdc.europa.eu/en/healthtopics/Documents/091015_Influenza_AH1N1_Situation_Report_0900hrs.pdf

European Centre for Disease Prevention and Control. (2009af, Oktober 27). ECDC Daily Update. Pandemic (H1N1) 2009. Available from: http://ecdc.europa.eu/en/healthtopics/Documents/091027_Influenza_AH1N1_Situation_Report_0900hrs.pdf

European Centre for Disease Prevention and Control. (2009ag, November 6). ECDC Risk Assessment. Pandemic H1N1 2009. Available from: http://ecdc.europa.eu/en/healthtopics/H1N1/Documents/1001_RA_091106.pdf

European Centre for Disease Prevention and Control. (2009ah, November 16). ECDC Daily Update. Pandemic (H1N1) 2009. Available from: http://www.ecdc.europa.eu/en/healthtopics/Documents/091116_Influenza_AH1N1_Situation_Report_0900hrs.pdf

European Centre for Disease Prevention and Control. (2009ai, Dezember 4). ECDC Daily Update. Pandemic (H1N1) 2009. Available from:

http://ecdc.europa.eu/en/healthtopics/Documents/091204_Influenza_AH1N1_Situation_Report_0900hrs.pdf

European Centre for Disease Prevention and Control. (2009aj, Dezember 11). ECDC Daily Update. Pandemic (H1N1) 2009. Available from: http://www.ecdc.europa.eu/en/healthtopics/Documents/091211_Influenza_AH1N1_Situation_Report_0900hrs.pdf

European Centre for Disease Prevention and Control. (2009ak, Dezember 11). Surveillance Report. Weekly influenza surveillance overview. Available from: http://ecdc.europa.eu/en/publications/Publications/091211_EISN_Weekly_Influenza_Surveillance_Overview.pdf

European Centre for Disease Prevention and Control. (2009al, Dezember 28). ECDC Daily Update.2009 influenza A (H1N1) pandemic. Available from: http://ecdc.europa.eu/en/healthtopics/Documents/091228_Influenza_AH1N1_Situation_Report_0900hrs.pdf

European Centre for Disease Prevention and Control. (2010a). European 2009 Influenza Pandemic Timeline. ECDC. Available from: http://ecdc.europa.eu/en/healthtopics/H1N1/Documents/110810_2009_pandemic_European_Timeline.pdf

European Centre for Disease Prevention and Control. (2010b, Januar 15). ECDC Daily Update. Pandemic (H1N1) 2009. Available from: http://www.ecdc.europa.eu/en/healthtopics/Documents/100115_Influenza_AH1N1_Situation_Report_0900hrs.pdf

European Centre for Disease Prevention and Control. (2010c, Januar 15). Surveillance Report. Weekly influenza surveillance overview. Available from: http://ecdc.europa.eu/en/publications/Publications/100115_EISN_Weekly_Influenza_Surveillance_Overview.pdf

European Centre for Disease Prevention and Control. (2010d, Februar 19). Surveillance Report. Weekly influenza surveillance overview. Available from: http://ecdc.europa.eu/en/publications/Publications/100219_EISN_Weekly_Influenza_Surveillance_Overview.pdf

European Centre for Disease Prevention and Control. (2010e, März 19). Surveillance Report. Weekly influenza surveillance overview. Available from:

http://ecdc.europa.eu/en/publications/Publications/100319_EISN_Weekly_Influenza_Surveillance_Overview.pdf

European Commission. (2009a). Commission Decision of 30 April 2009 amending Decision 2002/253/EC laying down case definitions for reporting communicable diseases to the Community network under Decision No 2119/98/EC of the European Parliament and of the Council. *Official Journal of the European Union*. Available from: http://eur-lex.europa.eu/LexUriServ/LexUriServ.do?uri=OJ:L:2009:110:0058:0059:EN:PDF

European Commission. (2009b, September 29). Commission paves the way for vaccinations for influenza pandemic (H1N1) 2009. Available from: http://europa.eu/rapid/pressReleasesAction.do?reference=IP/09/1384&format=HTML&aged=0&language=EN&guiLanguage=en

European Commission. (2009c, Oktober 7). Midday Express. News from the Communication Directorate General`s midday briefing. Available from: http://europa.eu/rapid/pressReleasesAction.do?reference=MEX/09/1007&format=HTML&aged=0&language=EN&guiLanguage=en

European Medicines Agency. (2009a, Mai 8). Press Release. European Medicines Agency gives guidance for use of antiviral medicines in case of a novel influenza A/H1N1 pandemic. Available from: http://www.ema.europa.eu/docs/en_GB/document_library/Press_release/2009/11/WC500011127.pdf

European Medicines Agency. (2009b, Oktober 23). Press Release. Meeting highlights from the Committee for Medicinal Products for Human Use, 19-22 October 2009. Available from: http://www.emea.europa.eu/docs/en_GB/document_library/Press_release/2009/11/WC500014317.pdf

European Medicines Agency. (2009c, November 20). Press Release. European Medicines Agency reaffirms efficacy and safety of H1N1 pandemic vaccines. Available from: http://www.emea.europa.eu/docs/en_GB/document_library/Press_release/2009/11/WC500015558.pdf

European Medicines Agency. (2010). Pandemic influenza pharmacovigilance updates. Available from: http://www.emea.europa.eu/ema/index.jsp?curl=pages/special_topics/general/general_content_000246.jsp&mid=WC0b01ac058004bf57

ECOM

Feufel, M. A., Antes, G., & Gigerenzer, G. (2010). Vom sicheren Umgang mit Unsicherheit: Was wir von der pandemischen Influenza (H1N1) 2009 lernen können, (53), 1283–1289. doi:10.1007/s00103-010-1165-1

Gilsdorf, A., & Poggensee, G. (2009). Influenza A(H1N1)v in Germany: The first 10.000 cases. *Euro Surveillance*, *14*(34). Available from: http://www.eurosurveillance.org/images/dynamic/EE/V14N34/art19318.pdf

Ginsberg, J., Mohebbi, M.H., Patel, R.S., Brammer, L., Smolinski, M.S. & Brilliant, L. (2008): Detecting influenza epidemics using search engine query data. *Nature* 457(7232), 1012-1014.

Greco, D., Stern, E., & Marks, G. (2011). Review of ECDC's response to the influenza pandemic 2009–2010. ECDC. Available from: http://ecdc.europa.eu/en/publications/Publications/241111COR_Pandemic_response.pdf

Hardelid, P., Andrews, N. ., Hoschler, K., Stanford, E., Baguelin, M., Waight, P., … Miller, E. (2010). Assessment of baseline age-specific antibody prevalence and incidence of infection to novel influenza A/H1N1 2009. *Health Technology Assessment*, *14*(55), 115–192.

Harder, K., Andersen, P. H., Baer, I., Nielsen, L., Ethelberg, S., Gliesmann, S., & Mølbak, K. (2011). Electronic real-time surveillance for influenza-like illness: experience from the 2009 influenza A(H1N1) pandemic in Denmark. *Euro Surveillance*, *16*(3). Available from: http://www.eurosurveillance.org/ViewArticle.aspx?ArticleId=19767

Health Protection Agency. (2009a, Mai 21). Update on confirmed swine flu cases. Available from: http://www.hpa.org.uk/NewsCentre/NationalPressReleases/2009PressReleases/090521Updateonconfirmedswineflucases/

Health Protection Agency. (2009b, Juli 1). HPA Weekly National Influenza Report. 01 July 2009 (Week 27). Available from: http://www.hpa.org.uk/webc/HPAwebFile/HPAweb_C/1246433639498

Health Protection Agency. (2009c, August 12). Method used to estimate new pandemic (H1N1) 2009 influenza cases in England in the week 3 August to 9 August 200. Available from: http://www.hpa.org.uk/webc/HPAwebFile/HPAweb_C/1250150839845

Health Protection Agency. (2009d, Dezember 2). Pandemic (H1N1) 2009 in England: an overview of initial epidemiological findings and implications for the second wave. Available from: http://www.hpa.org.uk/webc/HPAwebFile/HPAweb_C/1258560552857

Health Protection Agency. (2010a). Weekly epidemiological updates archive. Available from: http://www.hpa.org.uk/Topics/InfectiousDiseases/InfectionsAZ/PandemicInfluenza/H1N1PandemicArchive/SIEpidemiologicalData/SIEpidemiologicalReportsArchive/influswarchiveweeklyepireports/

Health Protection Agency. (2010b). Epidemiological report of pandemic (H1N1) 2009 in the UK. April 2009 – May 2010. Available from: http://www.hpa.org.uk/webc/HPAwebFile/HPAweb_C/1284475321350

Health Protection Agency. (2010c, März). The role of the Health Protection Agency in the 'containment' phase during the first wave of pandemic influenza in England in 2009. Available from: http://www.hpa.org.uk/webc/HPAwebFile/HPAweb_C/1274088320581

Health Protection Agency. (2012). International flu surveillance. Available from: http://www.hpa.org.uk/Topics/InfectiousDiseases/InfectionsAZ/SeasonalInfluenza/EpidemiologicalData/50influsInternationalflusurveillance/

Hilton, S., & Hunt, K. (2011). UK newspapers' representations of the 2009–10 outbreak of swine flu: one health scare not over-hyped by the media?. *Journal of epidemiology and community Health*, 65(10), 941-946.

Hine, D. D. (2010). The 2009 Influenza Pandemic. An independent review of the UK response to the 2009 influenza pandemic. Available from: http://webarchive.nationalarchives.gov.uk/+/http://www.cabinetoffice.gov.uk/media/416533/the2009influenzapandemic-review.pdf

Krause, G., Gilsdorf, A., Becker, J., Bradt, K., Dreweck, C., Gärtner, B., … Razum, O. (2010). Erster Erfahrungsaustausch zur H1N1-Pandemie in Deutschland 2009/2010. Bericht über einen Workshop am 22. und 23. März 2010 in Berlin, (53), 510–519. doi:10.1007/s00103-010-1074-3

Kyncl, J., Havlickova, M., Nagy, A., Jirincova, H., & Piskova, I. (2013). Early and unexpectedly severe start of influenza epidemic in the Czech Republic during

influenza season 2012-13. *Euro Surveillance*, *18*(6). Available from: http://www.eurosurveillance.org/ViewArticle.aspx?ArticleId=20396

Larrauri, A., Savulescu, C., Jiménez-Jorge, S., Pérez-Brena, P., Pozo, F., Casas, I., … de Mateo, S. (2011). Influenza pandemic (H1N1) 2009 activity during summer 2009. Effectiveness of the 2008-9 trivalent vaccine against pandemic influenza in Spain. *Gac Sanit.*, *25*(1), 23–28.

Larrauri Cámara, A., Jiménez-Jorge, S., Méndez, L. S., & de Mateo Ontañón, S. (2010). Vigilancia de la Pandemia de Gripe (H1N1) 2009 en ESPAÑA. *Revista Española de Salud Pública*, *84*(5), 569–588.

Louie, J. ., Acosta, M., Winter, K., Jean, C., Gavali, S., Schechter, R., … Hatch, D. (2009). Factors associated with death or hospitalization due to pandemic 2009 influenza A(H1N1) infection in California. *JAMA*, *302*(17), 1896–902.

Marcic, A., Dreesman, J., Liebl, B., Schlaich, C., Suckau, M., Sydow, W., & Wirtz, A. (2010). H1N1-Pandemie Maßnahmen und Erfahrungen auf Landesebene. *Bundesgesundheitsblatt*, *53*(12), 1257–1266. doi:10.1007/s00103-010-1164-2

Martin, T. (2010). Pandemie als kommunikative Herausforderung für die Bundesbehörden-ein Überblick über die Maßnahmen der Öffentlichkeitsarbeit der Bundesbehörden zur Neuen Grippe („Schweinegrippe“). *Bundesgesundheitsblatt*, *53*, 1304–1307.

Medicines and Healthcare products Regulatory Agency. (2009a, Juli 6). Swine flu - reporting suspected adverse reactions to Tamiflu, Relenza and future Swine flu H1N1 vaccines. Available from: http://www.mhra.gov.uk/home/groups/pl-p/documents/websiteresources/con051791.pdf

Medicines and Healthcare products Regulatory Agency. (2009b, November 5). UK Suspected Adverse Reaction Analysis. Swine Flu (H1N1) Vaccines. 5 November 2009. Available from: http://www.mhra.gov.uk/home/groups/pl-p/documents/websiteresources/con062636.pdf

Mereckiene, J., Cotter, S., Weber, J., Nicoll, A., D’Ancona, F., Lopalco, P., … the VENICE project gatekeepers group. (2012). Influenza A(H1N1)pdm09 vaccination policies and coverage in Europe. *Euro Surveillance*, *17*(4). Available from: http://www.eurosurveillance.org/ViewArticle.aspx?ArticleId=20064

Ministerio de Sanidad y Politica Social. (2009a). Gripe A. La prevención es la mejor medida. Available from: http://www.msssi.gob.es/campannas/campanas09/informacionGripeA.htm

Ministerio de Sanidad y Politica Social. (2009b, Juni 29). Informe diario de situación Nacional e Internacional. Gripe A/H1N1. Available from: http://www.msssi.gob.es/servCiudadanos/alertas/informesGripeA/090629.htm

Ministerio de Sanidad y Politica Social. (2009c, Juli 28). Cambio en el sistema de información sobre los casos de gripe A/ H1N1 en situación de pandemia en fase 6. Available from: http://www.msssi.gob.es/servCiudadanos/alertas/informesGripeA/090728.htm

Ministerio de Sanidad y Politica Social. (2009d, August). Protocolo de manejo de la Insuficiencia Respiratoria Aguda Grave en pacientes con Neumonía Viral Primaria por el nuevo virus de la Gripe A (H1N1) en UCI. Available from: http://www.msps.es/va/profesionales/saludPublica/gripeA/guiasProtocolosInf/pdf/ProtocoloGripeAenUCI.pdf

Ministerio de Sanidad y Politica Social. (2009e, September). Guia sobra la nueva Gripe para las familias. (Versión 2. Recomendaciones provisionales sujetas a actualización según se disponga de nueva información científica). Available from: http://www.msc.es/servCiudadanos/alertas/pdf/09-09-10_guia_gripe_A_Familias.pdf

Ministerio de Sanidad y Politica Social. (2009f, September). Criterios Generales de Actuación frente a la Gripe pandémica A (H1N1) en el Ámbito Escolar (Versión 2. Recomendaciones provisionales sujetas a actualización según se disponga de nueva información científica). Available from: http://www.msc.es/servCiudadanos/alertas/pdf/09-09-10_Recomendaciones_AmbitoEscolar.pdf

Ministerio de Sanidad y Politica Social. (2009g, September). Criterios Generales de Actuación frente a la Gripe pandémica A (H1N1) en Escuelas Infantiles y otros centros de atención a la primera infancia (Versión 1. Recomendaciones provisionales sujetas a actualización según se disponga de nueva información científica). Available from: http://www.msc.es/servCiudadanos/alertas/pdf/09-09-11-Guarderias.pdf

Ministerio de Sanidad y Politica Social. (2009h, September). Prevención y Tratamiento de la infección por el nuevo virus de la gripe A (H1N1) en la mujer embarazada con

especial atención al medio laboral y sanitario. (Versión 2. Recomendaciones provisionales sujetas a actualización según se disponga de nueva información científica). Available from: http://www.msc.es/profesionales/saludPublica/gripeA/guiasProtocolosInf/pdf/09-10-09_Embarazada.pdf

Ministerio de Sanidad y Politica Social. (2009i, September). Guía del manejo clínico de la neumonía adquirida en la comunidad en el adulto durante la pandemia por el nuevo virus influenza A(H1N1) (Versión 2. Recomendaciones provisionales sujetas a actualización según se disponga de nueva información científica). Available from: http://www.msc.es/profesionales/saludPublica/gripeA/guiasProtocolosInf/pdf/neumonia.pdf

Ministerio de Sanidad y Politica Social. (2009j, Oktober). Recomendaciones para profesionales de atención primaria sobre el manejo diagnóstico y terapéutico de la infección por el virus pandémico (H1N1) 2009 y la organización de la asistencia (Versión 1. Recomendaciones provisionales sujetas a actualización según se disponga de nueva información científica). Available from: http://www.msc.es/profesionales/saludPublica/gripeA/guiasProtocolosInf/pdf/09-12-02-atencionPrimaria.pdf

Ministerio de Sanidad y Politica Social. (2009k, Oktober). Recomendaciones para la prevención y el control de la infección en las residencias de personas mayores y otras modalidades de población institucionalizada ante el nuevo virus de la gripe pandémica (H1N1) 2009. Available from: http://www.msps.es/va/profesionales/saludPublica/gripeA/guiasProtocolosInf/pdf/09-12-02_ResidenciasPersonasMayores.pdf

Ministerio de Sanidad y Politica Social. (2009l, November). Swine Flu Vaccination 2009. Available from: http://www.informaciongripea.es/descargas/fichas/fase2/FICHA_VACUNACION_INGLES_baja.pdf

Ministerio de Sanidad y Politica Social. (2012). informaciongripea. Available from: http://www.facebook.com/informaciongripea

Ministerio de Sanidad, Politica Social e Igualdad. (2010a, November). Informe del analisis de las actuaciones en materia de vigilancia durante la pandemia de gripe por virus A (H1N1)2009. Available from:

http://www.mspsi.gob.es/profesionales/saludPublica/gripeA/docs/informeAnalisisNov2010.pdf

Ministerio de Sanidad, Politica Social e Igualdad. (2010b, Dezember). Anális de la actuación en materia de vacunas y antivirales durante la pandemia de Gripe por virus A (H1N1)2009. Available from: http://www.mspsi.gob.es/profesionales/saludPublica/gripeA/docs/informeSVAdic2010.pdf

Ministerio de Sanidad, Servicios Sociales e Igualdad. (2009). Medidas de Prevención y Control ante la Gripe A (H1N1) en los Centros Educativos. Available from: http://www.msc.es/servCiudadanos/alertas/recomendacionesCentrosEducativos.htm

Ministerstvo zdravotnictví ČR. (2009a). Pro cestovatele 21.5.09 - Aktualizavané Doporučení Ministerstva zdravotnictví pro cestovatele. Available from: http://pandemie.mzcr.cz/Pages/104-21509-aktualizavane-doporuceni-ministerstva-zdravotnictvi-pro-cestovatele.html

Ministerstvo zdravotnictví ČR. (2009b). Léky RELENZA – informace pro zdravotníky. Available from: http://pandemie.mzcr.cz/Pages/286-relenza-informace-pro-zdravotniky.html

Ministerstvo zdravotnictví ČR. (2009c). Vakcinační strategie. Available from: http://pandemie.mzcr.cz/Pages/402-vakcinacni-strategie.html

Ministerstvo zdravotnictví ČR. (2009d). Otázky a odpovědi 27.8.09 - Výskyt chřipkového viru A/H1N1 a Vy. Available from: http://pandemie.mzcr.cz/Pages/249-27809-vyskyt-chripkoveho-viru-ah1n1-a-vy.html

Ministerstvo zdravotnictví ČR. (2009e). Prevence a opatření při výskytu chřipky A(H1N1) – určeno pro pacienty. Available from: http://pandemie.mzcr.cz/Pages/124-prevence-a-opatreni-pri-vyskytu-chripky-ah1n1-urceno-pro-pacienty.html

Ministerstvo zdravotnictví ČR. (2009f). Léky Tamiflu – aktualizace údajů pro zdravotníky. Available from: http://pandemie.mzcr.cz/Pages/285-tamiflu-aktualizace-udaju-pro-zdravotniky.html

Ministerstvo zdravotnictví ČR. (2009g). Vakcíny a očkování Informační materiál zaslaný praktickým lékařům pro děti a dorost 16.11.2009. Available from: http://pandemie.mzcr.cz/Pages/364-informacni-material-zaslany-praktickym-lekarum-pro-deti-a-dorost-16112009.html

Ministerstvo zdravotnictví ČR. (2009h). Vakcíny a očkování Informační materiál zaslaný 20.11.2009 na lůžková zdravotnická zařízení k distribuce Tamiflu. Available from: http://pandemie.mzcr.cz/Pages/366-informacni-material-zaslany-20112009-na-luzkova-zdravotnicka-zarizeni-k-distribuce-tamiflu.html

Ministerstvo zdravotnictví ČR. (2009i). Vakcíny a očkování Informační materiál odeslaný 20.11.2009 na vakcinační centra. Available from: http://pandemie.mzcr.cz/Pages/367-informacni-material-odeslany-20112009-na-vakcinacni-centra.html

Ministerstvo zdravotnictví ČR. (2009j). Rozhodnutí a mimořádné opatření Rozhodnutí ze dne 25. 11. 2009, kterým se stanoví mimořádné opatření, které ukládá povinnost zdravotnickým zařízením (vakcinační centra) provést očkování pandemickou vakcínou Pandemrix. Available from: http://pandemie.mzcr.cz/Pages/409-rozhodnuti-ze-dne-25-11-2009-kterym-se-stanovi-mimoradne-opatreni-ktere-uklada-povinnost-zdravotnickym-zarizenim-vakcinacni-centra-provest-ockovani-pandemickou-vakcinou-pandemrix.html

Ministerstvo zdravotnictví ČR. (2009k). Otázky a odpovědi 9.12.09 Vybrané otázky a odpovědi týkající se vakcín a očkování v souvislosti s pandemií chřipky (H1N1) 2009. Available from: http://pandemie.mzcr.cz/Pages/434-91209-vybrane-otazky-a-odpovedi-tykajici-se-vakcin-a-ockovani-v-souvislosti-s-pandemii-chripky-h1n1-2009.html

Ministerstvo zdravotnictví ČR. (2010). Údaje k výskytu podezření na onemocnění Pandemic (H1N1). Available from: http://pandemie.mzcr.cz/Categories/134-udaje-k-vyskytu-podezreni-na-onemocneni-pandemic-h1n1.html

Mølbak, K., Widgren, K., Jensen, K., Ethelberg, S., Andersen, P., Christiansen, A., … Glismann, S. (2011). Burden of illness of the 2009 pandemic of influenza A (H1N1) in Denmark. *Vaccine*, *29*(S2), B63–B69.

National Board of Health. (2009a). Information to passengers. Novel flu virus. Available from: http://www.sst.dk/publ/Publ2009/CFF/influenza/SS_Nyinfluenza_flyer_UK_web.pdf

National Board of Health. (2009b). 7. tilfælde af influenza A (H1N1) i Danmark. Available from: http://www.sst.dk/Nyhedscenter/Nyheder/2009/Tilfaelde_7_ny_influenza.aspx

National Board of Health. (2009c). Protect yourself and others from Influenza A(H1N1). Available from: http://www.sst.dk/publ/Publ2009/CFF/influenza/Beskyt_dig_Influenza%20A_plakatUK.pdf

National Board of Health. (2009d). Influenza A(H1N1). If you become ill while in Denmark. Available from: http://www.sst.dk/publ/publ2010/CFF/Influenzavaccination/Influenzafolder_COP15.pdf

National Board of Health. (2009e, Mai 1). Tilfælde af Influenza A (H1N1) i Danmark. Available from: http://www.sst.dk/Nyhedscenter/Nyheder/2009/FoersteTilfaeldeNyInfluenzaDK.aspx

National Board of Health. (2009f, Mai 18). Ny influenza A (H1N1): Sundhedsstyrelsen fraråder ikke længere rejse til Mexico. Available from: http://www.sst.dk/Nyhedscenter/Nyheder/2009/Rejserestriktioner_Mexico.aspx

National Board of Health. (2009g, Juni 11). WHO hæver influenzaniveau til fase 6 - pandemi. Available from: http://www.sst.dk/Nyhedscenter/Nyheder/2009/WHO%20fase%206.aspx

National Board of Health. (2009h, Juli 6). Ændret strategi for håndtering af influenza A (H1N1). Available from: http://www.sst.dk/Nyhedscenter/Nyheder/2009/Aendret%20strategi.aspx

National Board of Health. (2009i, September 3). Dansk statsborger, som døde i Norge, havde Influenza A (H1N1). Available from: http://www.sst.dk/Nyhedscenter/Nyheder/2009/Dansker%20doed%20af%20influenza.aspx

National Board of Health. (2009j, September 30). Vejledning til læger og andet sundhedspersonale om influenza A (H1N1)v. Available from: https://www.retsinformation.dk/Forms/R0710.aspx?id=127454

National Board of Health. (2009k, Oktober 1). Ny vejledning om håndtering af influenza A (H1N1). Available from: http://www.sst.dk/Nyhedscenter/Nyheder/2009/Influenza_ny_vejledn_haandt.aspx

National Board of Health. (2009l, Oktober 19). Bestil tid til influenza A (H1N1) vaccination. Available from: http://www.sst.dk/Nyhedscenter/Nyheder/2009/Vaccination_bestil_tid_65.aspx

National Board of Health. (2009m, Oktober 23). Justeringer i anbefalinger for vaccination mod influenza A (H1N1) i Danmark. Available from: http://www.sst.dk/Nyhedscenter/Nyheder/2009/Anbefalinger_vaccination_risikogrupper23okt.aspx

National Board of Health. (2009n, Oktober 23). Anbefalinger for vaccination af personer i risiko for alvorlig sygdom pga. influenza A(H1N1)v infektion. Available from: http://www.sst.dk/~/media/Sundhed%20og%20forebyggelse/Smitsomme%20sygdomme/Influenza/Vaccination_lister/Anbefalinger_vaccination_risikogrupper23okt.ashx

National Board of Health. (2009o, November 30). Influenza A-information på fremmedsprog. Available from: http://www.sst.dk/Nyhedscenter/Nyheder/2009/Influenza_information_fremmedsprog.aspx

National Board of Health. (2009p, Dezember 2). Justering af anbefalinger vedrørende vaccination mod influenza A(H1N1). Available from: http://www.sst.dk/Nyhedscenter/Nyheder/2009/Justering_vaccine_anbefalinger.aspx

National Board of Health. (2009q, Dezember 9). Fem dødsfald med influenza A (H1N1). Available from: http://www.sst.dk/Nyhedscenter/Nyheder/2009/Doedsfald_8_8dec.aspx

National Board of Health. (2009r, Dezember 17). Sundhedsstyrelsens influenza A hotline stilles i bero. Available from: http://www.sst.dk/Nyhedscenter/Nyheder/2009/Hotline_bero_influenza17dec.aspx

National Board of Health. (2010, Februar 12). Gratis influenza A-vaccine til personer uden for risikogruppen. Available from: http://www.sst.dk/Nyhedscenter/Nyheder/2010/Overskydende_vaccine.aspx

Nicoll, A., & Coulombier, D. (2009). Europe's initial experience with pandemic (H1N1) 2009 - mitigation and delaying policies and practices. *Euro Surveillance*, *14*(29).

Available from: http://www.eurosurveillance.org/ViewArticle.aspx?ArticleId=19279

O'Flanagan, D., Cotter, S., & Mereckiene, J. (2011). Pandemic A(H1N1) 2009 Influenza Vaccination Survey, Influenza season 2009/2010.VENICE II Consortium August 2010-April 2011. Available from: http://venice.cineca.org/Final_Report_VENICE_Pandemic_Influenza_2009.pdf

Paul-Ehrlich-Institute. (2009, September 4). Fachliche Information für Ärzte und Apotheker: Pandemie-Impfstoffe in der Schwangerschaft - Sicherheitsaspekte. Available from: http://www.pei.de/cln_101/SharedDocs/Downloads/fachkreise/090309-pandemieimpfstoffe-schwangerschaft,templateId=raw,property=publicationFile.pdf/090309-pandemieimpfstoffe-schwangerschaft.pdf

Pebody, R. G., McLean, E., Zhao, H., Cleary, P., Bracebridge, S., Foster, K., … Watson, J. M. (2010). Pandemic Influenza A (H1N1) 2009 and mortality in the United Kingdom: risk factors for death, April 2009 to March 2010. *Euro Surveillance*, *15*(20). Available from: http://www.eurosurveillance.org/images/dynamic/EE/V15N20/art19571.pdf

Robert Koch-Institute. (2009a). RKI-Ratgeber Infektionskrankheiten – Merkblätter für Ärzte: Influenza. *Epidemiologisches Bulletin*, *2009*(43), 438–451.

Robert Koch-Institute. (2009b). RKI - Influenzapandemie (H1N1) 2009 - Archiv der Situationseinschätzungen. Available from: http://www.rki.de/DE/Content/InfAZ/I/Influenza/IPV/Archiv-Situation/Tab-Situation.html?nn=2370464&cms_gtp=2399050_list%253D6 (20.10.2011)

Robert Koch-Institute. (2009c). STIKO-Empfehlung zur Impfung gegen die Neue Influenza A (H1N1). *Epidemiologisches Bulletin*, *2009*(41), 403–424.

Robert Koch-Institute. (2009d). Influenza-Wochenbericht für die Woche 26 (20.06. – 26.06.2009). Available from: http://influenza.rki.de/Wochenberichte/2008_2009/2009-26.pdf

Robert Koch-Institute. (2009e). Zur Schließung von Kindergemeinschaftseinrichtungen im Zusammenhang mit Neuer Influenza A/H1N1. *Epidemiologisches Bulletin*, *2009*(46), 475–476.

Robert Koch-Institute. (2009f). Mitteilung der Ständigen Impfkommission (STIKO) am Robert Koch-Institut. Impfung gegen die Neue Influenza A (H1N1). Erneute Bewertung der Daten am 24.11.2009. *Epidemiologisches Bulletin*, *2009*(50), 513–519.

Robert Koch-Institute. (2009g). Ergänzende Hinweise des Paul-Ehrlich-Instituts und des Robert Koch-Instituts zur Impfung gegen die Neue Influenza A (H1N1). *Epidemiologisches Bulletin*, *2009*(50), 519–520.

Robert Koch-Institute. (2009h, April 29). Situation in Deutschland - 29.04.2009. Available from: http://www.rki.de/DE/Content/InfAZ/I/Influenza/IPV/Archiv-Situation/Schweineinfluenza_Situation-090429.html

Robert Koch-Institute. (2009i, Juli). Influenza Typ A/H1N1. Available from: http://www.bundesaerztekammer.de/downloads/InfluenzaAH1N1.pdf

Robert Koch-Institute. (2009j, September 28). Situation in Deutschland- 28.09.2009. Available from: http://www.rki.de/DE/Content/InfAZ/I/Influenza/IPV/Archiv-Situation/Schweineinfluenza_Situation-090928.html

Robert Koch-Institute. (2010a). Rückblick: Epidemiologie und Infektionsschutz im zeitlichen Verlauf der Influenzapandemie (H1N1) 2009. *Epidemiologisches Bulletin*, *21*, 191–197.

Robert Koch-Institute. (2010b). Repräsentative telefonische Erhebung zur Impfung gegen die pandemische Influenza (H1N1) 2009. Ergebnisse aus Befragungen bis April 2010, (25), 237–238.

Robert Koch-Institute, & Bundeszentrale für gesundheitliche Aufklärung. (2009, März). Wir gegen Viren. Available from: http://www.wir-gegen-viren.de/ (16.05.2012)

Robert Koch-Institute, & Paul-Ehrlich-Institute. (2009, Oktober). Die Impfung zum Schutz vor der Neuen Influenza A (H1N1) – Hinweise für das medizinische Personal. Available from: http://www.dkgev.de/dkg.php/aid/6630/cat/43

Robert Koch-Institute. Arbeitsgemeinschaft Influenza. (2010). Wochenberichte der AGI. Available from: http://influenza.rki.de/Wochenberichte.aspx

Rubin, G., Amlot, R., Page, L., & Wessely, S. (2009). Public perceptions, anxiety,and behaviour change in relation to the swine flu outbreak: cross sectional telephone survey. *BMJ*, *339*. doi:10.1136

Rubin, G., Potts, H., & Michie, S. (2010). The impact of communications about swine flu (influenza A H1N1v) on public responses to the outbreak: results from 36 national telephone surveys in the UK. *Health Technology Assessment*, *14*(34), 165–248.

Santa-Olalla Peralta, P., Cortes García, M., Martínez Sánchez, E. V., Nogareda Moreno, F., Limia Sánchez, A., Pachón del Amo, I., & Sierra Moros, J. (2010). Vigilancia individualizada de los casos iniciales de infección por gripe pandémica (H1N1) 2009 en España, abril-junio 2009. *Revista Española de Salud Pública*, *84*(5), 529–546.

Santa-Olalla Peralta, P., Cortes García, M., Vicente-Herrero, M., Castrillo-Villamandos, C., Arias-Bohigas, P., Pachon-del Amo, I., … on behalf of the Surveillance Group for New Influenza A(H1N1) Virus Investigation and Control Team in Spain. (2010). Risk factors for disease severity among hospitalised patients with 2009 pandemic influenza A (H1N1) in Spain, April – December 2009. *Euro Surveillance*, *15*(38). Available from: http://www.eurosurveillance.org/ViewArticle.aspx?ArticleId=19667

Schaade, L., Reuß, A., Haas, W., & Krause, G. (2010). Pandemieplanung. Was haben wir aus der Pandemie (H1N1) 2009 gelernt? *Bundesgesundheitsblatt*, *53*, 1277–1282.

Schaberg, T., & Burger, R. (2010). Die Influenza-Pandemie der Saison 2009/2010. *Pneumologie*, *64*, 755–768.

Sekkides, O. (2010). Pandemic influenza-a timeline. *The Lancet Infectious Diseases*, *10*(10), 663.

Sethi, M. & Pebody, R. (2010a): Pandemic H1N1 (Swine Flu) and Seasonal Influenza Vaccine Uptake amongst Frontline Healthcare Workers in England 2009/10. Available from: https://www.gov.uk/government/uploads/system/uploads/attachment_data/file/215976/dh_121015.pdf (08.08.2014)

Sethi, M. & Pebody, R. (2010b): Pandemic H1N1 (Swine) Influenza Vaccine Uptake amongst Patient Groups in Primary Care in England 2009/2010. Available from: https://www.gov.uk/government/uploads/system/uploads/attachment_data/file/215977/dh_121014.pdf (08.08.2014)

Sierra Moros, J., Vázquez Torres, M., Santa-Olalla Peralta, P., Limia Sánchez, A., Crtes Garcia, M., & Pachón del Amo, I. (2010). Actividades de vigilancia epidemiológica

durante la pandemia de Gripe (H1N1) 2009 en España. Reflexiones un año después. *Revista Española de Salud Pública*, *84*(5), 463–479.

Stein, M. L., van Vliet, J. A., & Timen, A. (2011). Chronological overview of the 2009/2010 H1N1 influenza pandemic and the response of the Centre for Infectious Disease Control RIVM. Available from: http://www.rivm.nl/bibliotheek/rapporten/215011006.pdf

Stern, E. K., Young, S., Amlôt, R., Blake, A., Dacey, G., Lightfoot, N., … Thakrar, N. (2010, August 25). Assessment Report on EU-wide Pandemic Vaccine Strategies. Available from: http://ec.europa.eu/health/communicable_diseases/docs/assessment_vaccine_en.pdf

Surveillance Group for New Influenza A(H1N1) Virus Investigation and Control in Spain. (2009). New influenza A(H1N1) virus infections in Spain, April-May 2009. *Euro Surveillance*, *14*(19). Available from: http://www.eurosurveillance.org/ViewArticle.aspx?ArticleId=19209

The Gallup Organization. (2010, March). Flash Eurobarometer 287. Eurobarometer on Influenza H1N1. Available from: http://ec.europa.eu/public_opinion/flash/fl_287_en.pdf

The Secretary of State for Health. (2010, April 6). Swne Flu Pandemic Response. Available from: http://www.publications.parliament.uk/pa/cm200910/cmhansrd/cm100406/wmstext/100406m0002.htm#10040611000118

Tomášková, H., Boháčová, S. & Šlachtová, H. (2012): Attitudes of the medical students from two Czech universities to pandemic flu A (H1N1) 2009 and to influenza vaccination. *Central European journal of public health* 20(3), 215-218.

Venice II. (2011, April). O'Flanagan D, Cotter S and Mereckiene J. Pandemic A(H1N1) 2009 Influenza Vaccination Survey, Influenza season 2009/2010. Available from: http://venice.cineca.org/Final_Report_VENICE_Pandemic_Influenza_2009.pdf

Walter, Böhmer, M. M., an der Heiden, M., Reiter, S., Krause, G., & Wichmann, O. (2011). Monitoring pandemic influenza A(H1N1) vaccination coverage in Germany 2009/10 – Results from thirteen consecutive cross-sectional surveys. *Vaccine*, *2011*(29), 4008–4012.

Walter, D., Böhmer, M. M., Reiter, S., Krause, G., & Wichmann, O. (2012). Risk perception and information-seeking behaviour during the 2009/10 influenza A(H1N1)pdm09 pandemic in Germany. *Euro Surveillance*, *17*(13). Available from: http://www.eurosurveillance.org/ViewArticle.aspx?ArticleId=20131

World Health Organization. (2005). WHO Outbreak Communication. WHO Handbook for Journlists: Influenza Pandemic. Available from: http://www.who.int/csr/don/Handbook_influenza_pandemic_dec05.pdf

World Health Organization. (2009a). New influenza A (H1N1) virus: WHO guidance on public health measures, 11 June 2009. *Weekly epidemiological record*, *84*(26), 261–268.

World Health Organization. (2009b, April 24). Influenza-like illness in the United States and Mexico. *Global Alert and Response (GAR). Disease Outbreak News*. Available from: http://www.who.int/csr/don/2009_04_24/en/index.html

World Health Organization. (2009c, April 25). Swine influenza. *Media Centre*. Available from: http://www.who.int/mediacentre/news/statements/2009/h1n1_20090425/en/ (12.01.2012)

World Health Organization. (2009d, April 27). Swine influenza. *Media Centre*. Available from:http://www.who.int/mediacentre/news/statements/2009/h1n1_20090427/en/index.html (22.02. 2012)

World Health Organization. (2009e, April 29). Influenza A(H1N1). *Media Centre*. Available from:http://www.who.int/mediacentre/news/statements/2009/h1n1_20090429/en/index.html (22.01.2012)

World Health Organization. (2009f, Mai 20). Summary report of a High-Level Consultation: new influenza A (H1N1). Geneva, 18 May 2009. Available from: http://www.who.int/csr/resources/publications/swineflu/High_Level_Consultation_18_May_2009.pdf

World Health Organization. (2009g, Juni). Patient Care Checklist. Available from:http://www.who.int/csr/resources/publications/swineflu/ah1n1_checklist.pdf

World Health Organization. (2009h, Juni 11). World now at the start of 2009 influenza pandemic. Available from:

http://www.who.int/mediacentre/news/statements/2009/h1n1_pandemic_phase6_20090611/en/index.html

World Health Organization. (2009i, Juni 11). What is pahse 6? Available from: http://www.who.int/csr/disease/swineflu/frequently_asked_questions/levels_pandemic_alert/en/index.html

World Health Organization. (2009j, Juli 13). WHO recommendations on pandemic (H1N1) 2009 vaccines. Available from: http://www.who.int/csr/disease/swineflu/notes/h1n1_vaccine_20090713/en/index.html

World Health Organization. (2009k, Juli 16). Changes in reporting requirements for pandemic (H1N1) 2009 virus infection. Available from: http://www.who.int/csr/disease/swineflu/notes/h1n1_surveillance_20090710/en/

World Health Organization. (2009l, August 6). Safety of pandemic vaccines. Pandemic (H1N1) 2009 briefing note 6. Available from: http://www.who.int/csr/disease/swineflu/notes/h1n1_safety_vaccines_20090805/en/index.html

World Health Organization. (2009m, September 18). Pandemic (H1N1) 2009 - update 66. Available from: http://www.who.int/csr/disease/swineflu/laboratory18_09_2009/en/

World Health Organization. (2009n, September 24). Director-General Statement following the fifth meeting of the Emergency Committee. Available from: http://www.who.int/csr/disease/swineflu/5th_meeting_ihr/en/index.html

World Health Organization. (2009o, November 19). Safety of pandemic vaccines. Available from: http://www.who.int/csr/disease/swineflu/notes/briefing_20091119/en/index.html

World Health Organization. (2009p, November 26). Director-General statement following the sixth meeting of the Emergency Committee. Available from: http://www.who.int/csr/disease/swineflu/6th_meeting_ihr/en/index.html

World Health Organization. (2009q, Dezember 22). Comparing deaths from pandemic and seasonal influenza. Pandemic (H1N1) 2009 briefing note 20. Available from:

http://www.who.int/csr/disease/swineflu/notes/briefing_20091222/en/index.html

World Health Organization. (2010a, Februar 24). Director-General statement following the seventh meeting of the Emergency Committee. Available from: http://www.who.int/csr/disease/swineflu/7th_meeting_ihr/en/index.html

World Health Organization. (2010b, August 6). Pandemic (H1N1) 2009 - update 112. Available from: http://www.who.int/csr/don/2010_08_06/en/index.html

World Health Organization. (2010c, August 10). H1N1 in post-pandemic period. Available from: http://www.who.int/mediacentre/news/statements/2010/h1n1_vpc_20100810/en/index.html

World Health Organization. (2011). Implementation of the International Health Regulations (2005) Report of the Review Committee on the Functioning of the International Health Regulations (2005) in relation to Pandemic (H1N1) 2009. Available from: http://apps.who.int/gb/ebwha/pdf_files/WHA64/A64_10-en.pdf

World Health Organization. (2012). Current WHO phase of pandemic alert (avian influenza H5N1). Available from: http://www.who.int/influenza/preparedness/pandemic/h5n1phase/en/
